# Energy stress-induced circDDX21 promotes glycolysis and facilitates hepatocellular carcinogenesis

**DOI:** 10.1038/s41419-024-06743-1

**Published:** 2024-05-21

**Authors:** Jingjing Luo, Yang Yang, Guang Zhang, Debao Fang, Kaiyue Liu, Yide Mei, Fang Wang

**Affiliations:** 1 The First Affiliated Hospital of USTC, Center for Advanced Interdisciplinary Science and Biomedicine of IHM, Division of Life Sciences and Medicine, University of Science and Technology of China, Hefei, Anhui China; 2 School of Basic Medical Sciences, Anhui Medical University, Hefei, Anhui China

**Keywords:** Cell growth, Cancer metabolism

## Abstract

Cancer cells undergo metabolic reprogramming in response to hostile microenvironments, such as energy stress; however, the underlying mechanisms remain largely unclear. It is also unknown whether energy stress-responsive circular RNA (circRNA) is involved in the regulation of glucose metabolism. Here we report that circDDX21 is upregulated in response to glucose deprivation by the transcription factor c-Myc. Functionally, circDDX21 is shown to promote glycolysis by increasing PGAM1 expression. Mechanistically, circDDX21 interacts with the RNA binding protein PABPC1, disrupting its association with the ubiquitin E3 ligase MKRN3. This disassociation attenuates MKRN3-mediated PABPC1 ubiquitination and enhances the binding of PABPC1 to PGAM1 mRNA, thereby leading to PGAM1 mRNA stabilization. The ability of the circDDX21-PGAM1 axis to promote hepatocellular carcinogenesis is validated in a xenograft mouse model. Additionally, in clinical hepatocellular carcinoma tissues, there is a positive correlation between circDDX21 and PGAM1 expression. These findings establish circDDX21 as an important regulator of glycolysis and suggest circDDX21 as a potential therapeutic target for hepatocellular carcinoma.

## Introduction

Metabolic reprogramming is a crucial hallmark of cancer cells [1]. In comparison to their healthy counterparts, cancer cells exhibit an aberrant alteration in glucose metabolism, known as the Warburg effect. This effect is characterized by enhanced glycolysis and reduced oxidative phosphorylation, even in the presence of sufficient oxygen [2]. The Warburg effect, along with increased glucose uptake, not only enables cancer cells to meet their high energetic and biosynthetic demands but also creates an acidic environment, providing a growth advantage for tumors [3, 4]. However, during rapid tumor growth, nutrients such as glucose are often limited, which requires cancer cells to reprogram their metabolism for survival and proliferation [5, 6].

It has been well established that glycolysis plays a pivotal role in both cancer initiation and progression [7, 8]. The mechanisms responsible for increased glycolysis in cancer cells include upregulated expression and enhanced activity of several glycolytic enzymes [9, 10]. Among these glycolytic enzymes, phosphoglycerate mutase 1 (PGAM1) is a rate-limiting enzyme that catalyzes the reversible conversion between 3-phosphoglycerate (3-PG) and 2-phosphoglycerate (2-PG) [11]. PGAM1 is frequently upregulated in various human cancers, including breast cancer, lung cancer, and hepatocellular carcinoma [12–14]. Overexpression of PGAM1 promotes cancer progression, whereas inhibition of PGAM1 using shRNAs or inhibitors effectively suppresses cancer progression [15–19]. Given the importance of PGAM1 in cancer progression, it is not surprising that PGAM1 is intricately regulated by multiple factors at the transcriptional, translational, and post-translational levels [20–24]. However, the molecular mechanisms underlying the dysregulated expression of PGAM1 in cancer cells remain largely unclear.

The human genome transcribes a large number of noncoding RNAs (ncRNAs), including long noncoding RNAs (lncRNAs) and circular RNAs (circRNAs) [25, 26]. LncRNAs have been reported to play important roles in the regulation of glycolysis under energy stress conditions [27–30]. For instance, the glucose deprivation-induced lncRNA GLCC1 promotes glycolysis and facilitates colorectal carcinogenesis by stabilizing c-Myc [31]. CircRNAs, another important class of ncRNAs, are covalently closed RNA molecules generated by back-splicing of pre-mRNAs [32, 33]. Through interactions with DNA, RNA, or proteins, circRNAs can influence gene transcription, mRNA stability, and protein translation, thereby regulating gene expression at different levels [34, 35]. CircRNAs have been functionally implicated in the regulation of diverse biological processes, including cell proliferation, cell cycle progression, and cellular metabolism. Notably, a number of circRNAs have been reported to participate in the metabolic reprogramming of glycolysis in cancer cells [36–40]. Moreover, dysregulated expression of circRNAs has been associated with many human diseases including cancer [41–43]. However, it remains unknown whether circRNA is involved in the metabolic alteration of cancer cells in response to energy stress conditions in the tumor microenvironment. More specifically, it has not been characterized whether energy stress-responsive circRNAs can couple glycolysis with tumorigenesis.

In the present study, we identified circDDX21 as a glucose deprivation-induced circRNA. Functionally, circDDX21 promotes glycolysis by increasing PGAM1 expression. Mechanistically, circDDX21 increases the binding of PABPC1 to PGAM1 mRNA by suppressing MKRN3-mediated PABPC1 ubiquitination, thereby enhancing the mRNA stability of PGAM1. Furthermore, circDDX21 functions as an oncogenic circRNA to promote the progression of hepatocellular carcinoma. Collectively, these findings suggest that glucose deprivation-responsive circDDX21 plays an important role in the regulation of glycolysis and reveal a critical role for circDDX21 in promoting hepatocellular carcinogenesis.

## Materials and methods

### Reagents and antibodies

The following reagents were used in this study: lipofectamine 2000 (11668019; Invitrogen), polybrene (TR-1003; Sigma-Aldrich), actinomycin D (S8964; Selleck), DAPI (H1200; Vector Laboratories), complete EDTA-free protease inhibitor cocktail (K1007; APExBIO), protein A/G agarose beads (20421; Thermo Fisher Scientific), anti-Flag M2 affinity agarose beads (A2220; Sigma), and streptavidin agarose resin (20349; Thermo Fisher Scientific).

The following antibodies were used in this study: anti-HK2 (22029-1-AP), anti-GPI (15171-1-AP), anti-ALDOB (18065-1-AP), anti-PGK1 (17811-1-AP), anti-PGAM1 (16126-1-AP), anti-ENO1 (11204-1-AP), anti-LDHA (19987-1-AP), and PABPC1 (10970-1-AP) antibodies were purchased from Proteintech. Anti-GFP (sc-9996), anti-β-actin (sc-47778), and anti-GAPDH (sc-166545) antibodies were purchased from Santa Cruz Biotechnology. Anti-c-Myc (9402 S), anti-PKM2 (4053 S), and anti-ubiquitin (3936 S) antibodies were purchased from Cell Signaling Technology. Anti-PFKL (ab181064) antibody was purchased from Abcam. Anti-Flag (F3165) and anti-MKRN3 (HPA029494) antibodies were purchased from Sigma-Aldrich. Horseradish peroxidase-conjugated secondary antibodies against rabbit (111-035-144) and mouse (115-035-062) were purchased from Jackson ImmunoResearch.

### Cell lines and tissues

The human hepatocellular carcinoma cell lines HepG2 and PLC were purchased from ATCC. These cells were cultured in Dulbecco’s modified Eagle’s medium (DMEM) (Gibco) supplemented with 10% FBS and antibiotics. All cell lines were maintained at 37 °C in an incubator with 5% CO2. Mycoplasma contamination of all cell lines was routinely monitored, and mycoplasma-free cells were used for experiments. Human liver tissue microarrays were obtained from Outdo Biotech (Shanghai, China).

### Production and infection of lentivirus

For overexpression of the indicated proteins or RNAs, HEK293T cells were transfected with pCDH-based or pSin-based constructs together with pMD2.G and psPAX2 at a ratio of 2:1:2. For shRNA-mediated knockdown of the indicated proteins or RNAs, HEK293T cells were transfected with shRNAs (cloned in PLKO.1) together with pREV, pGag, and pVSVG plasmids at a ratio of 2:2:2:1. For sgRNA-mediated knockdown of MKRN3, HEK293T cells were transfected with sgRNA (cloned in lentiCRISPRv2) together with psPAX2 and pVSVG at a ratio of 4:2:1. Forty-eight hours after transfection, the culture medium containing lentivirus particles was harvested and used for infection. Target cells were infected in the presence of 10 μg/mL polybrene to improve lentivirus transduction efficiency. To avoid off-target effects, two different circDDX21-targeting shRNAs (sh#1 and sh#2) were used in this study. The shRNA and sgRNA target sequences used in the study are listed in Supplementary Table 2.

### RNA extraction and RNase R treatment

Total RNA from the indicated cells was isolated using TRIzol reagent (Thermo Fisher Scientific) following the manufacturer’s instructions. RNA samples from PLC and HepG2 cells were incubated with 3 U/mg of RNase R at 37 °C for 10 min and 30 min, respectively, followed by RNA purification using a phenol-chloroform extraction method.

### Real-time RT-PCR

500 ng of total RNA was used for cDNA synthesis using HiScript III RT SuperMix (Vazyme) following the manufacturer’s instructions. Real-time PCR was performed using SYBR Green Master Mix (Vazyme) and analyzed with the StepOnePlus^TM^ real-time PCR system (Thermo Fisher Scientific). The primer sequences are shown in Supplementary Table 2.

### RNA fluorescence in situ hybridization (FISH)

RNA FISH was performed with an in vitro transcribed Alexa Fluor^®^546-labeled antisense probe targeting the back-splicing junction region of circDDX21 (ULYSIS^®^Nucleic Acid Labeling Kit, Thermo Fisher Scientific). HepG2 cells seeded on coverslips were fixed with 4% paraformaldehyde and permeabilized with 0.1% Triton X-100. Cells were then hybridized with the labeled RNA probe in hybridization buffer (50% formamide, 10% dextran sulfate, 2 × SSC, 1 mg/ml yeast transfer RNA, and 1 mg/ml sheared salmon sperm DNA) at 42 °C overnight. After washing with 50% formamide and 2 × SSC buffer, cells were counterstained with DAPI to visualize nuclei, and images were acquired by Leica DMI6000 B microscope. The RNA probe sequence is shown in Supplementary Table 2.

### Cytoplasmic/nuclear fractionation

HepG2 (1 × 10^6^) cells were lysed in 100 μL lysis buffer (20 mM HEPES, 10 mM KCl, 2 mM MgCl2, 0.5% NP-40, and RNase inhibitor) on ice for 20 min. After centrifugation at 5000 × *g* at 4 °C for 5 min, the supernatant was collected as the cytoplasmic fraction. The nuclei pellets were washed three times with PBS and then collected as the nuclear fraction.

### Luciferase reporter assay

To evaluate the effect of c-Myc on circDDX21 expression, HepG2 cells with knockdown or overexpression of c-Myc were transfected with pGL3, pGL3-BS1, pGL3-BS2, pGL3-BS3, or pGL3-BS3 mut plus Renilla luciferase plasmid. To examine the effect of circDDX21 on the PGAM1 3′-UTR, HepG2 cells with knockdown or overexpression of circDDX21 were transfected with the psiCHECK2-PGAM1 3′-UTR. Twenty-four hours after transfection, firefly and Renilla luciferase activities were measured by the Dual-Luciferase Reporter Assay System (Promega).

### ChIP assay

HepG2 cells were cross-linked with 1% formaldehyde for 10 min. The ChIP assay was then performed using the ChIP Assay Kit (Beyotime) with anti-c-Myc antibody or normal rabbit IgG according to the manufacturer’s instructions. The bound DNA fragments were analyzed by PCR using the primers in Supplementary Table 2.

### Biotin pull-down assay

HepG2 cells grown on a 10-cm dish were collected and lysed on ice for 20 min in 1 ml pull-down buffer (25 mM Tris, pH 7.4, 0.5% NP-40, 0.5 mM dithiothreitol, and 150 mM KCl) supplemented with protease inhibitor cocktail and RNase inhibitor. Cell lysates were incubated with sense or antisense biotin-labeled DNA oligomers corresponding to circDDX21 at 4 °C for 4 h. Streptavidin agarose beads (Thermo Fisher Scientific) were then added to isolate the RNA-protein complexes, followed by RT-PCR and western blot analyses.

### RNA immunoprecipitation (RIP)

HepG2 cells were lysed in RIPA buffer (50 mM Tris, pH 7.4, 150 mM NaCl, 1% NP-40, 0.5% sodium deoxycholate, 0.1% SDS, and 1 mM EDTA) supplemented with protease inhibitor cocktail, RNase inhibitor, and DNase I. Cell lysates were incubated with anti-Flag M2 beads at 4 °C for 4 h or with anti-PABPC1 antibody-coated protein A/G beads at 4 °C for 8 h. The immunocomplexes were eluted from the beads using elution buffer (50 mM Tris-HCl, pH 8.0, 1% SDS, and 10 mM EDTA) at 65 °C for 10 min. The purified RNAs and proteins from the eluted immunocomplexes were then subjected to RT-PCR and western blot analyses, respectively.

### In vitro circRNA cyclization

In vitro cyclization of linear RNA was performed as previously described [44]. The linear sense and antisense RNAs corresponding to circDDX21 were first synthesized using the MaxiScript T7 Kit (Ambion) according to the manufacturer’s instructions. The DNA templates were obtained by PCR using forward and reverse primers containing the T7 RNA polymerase promoter sequence. The in vitro transcribed linear RNA was incubated with DNA splints (molar ratio = 1:1.5) at 90 °C for 2 min before cooling the sample to room temperature. Ligation to form circRNA was then performed for 1 h at room temperature with T4 DNA ligase (Takara), followed by RNase R and DNase I treatment at 37 °C for 30 min. CircRNA was then purified using a phenol-chloroform extraction method. Primer and DNA splint sequences are shown in Supplementary Table 2.

### In vivo ubiquitination assay

HepG2 cells transduced with the indicated lentiviruses were lysed in denaturing buffer (150 mM Tris-HCl, pH 8.0, 1% SDS, and 30% glycerol) and boiled for 10 min. Cell lysates were then diluted 10 times with dilution buffer (50 mM Tris-HCl, pH 8.0, 150 mM NaCl, 0.5% NP-40, 1 × protease inhibitor cocktail), followed by immunoprecipitation with anti-PABPC1 antibody. The input and immunoprecipitates were then analyzed by western blotting with an anti-ubiquitin antibody.

### Extracellular acidification rate (ECAR) measurement

The extracellular acidification rate (ECAR) of the indicated cells was measured using the Seahorse XF Glycolysis Stress Test Kit (Agilent Technologies) according to the manufacturer’s instructions. Briefly, 2.5 × 10^4^ cells/well were seeded in a 96-well XF microplate and incubated for 24 h. The ECAR was then measured with an XFe96 Extracellular Flux Analyzer (Agilent Technologies) in XF base medium (pH 7.4) containing 1 mM glutamine following sequential additions of glucose (10 mM), oligomycin (1 mM), and 2-DG (50 mM).

### Colony formation assay

For the colony formation assay, 3 × 10^3^ HepG2 cells transduced with the indicated lentiviruses were seeded in a 6-well plate. Fourteen days later, colonies formed on the plate were fixed with 4% paraformaldehyde and stained with crystal violet. The number of clones was counted and analyzed using ImageJ software. Data shown are mean ± SD from three independent biological replicates.

### LC-MS-based metabolite analysis

For analysis of metabolites, 3 × 10^6^ HepG2 cells transduced with the indicated lentiviruses were cultured in glucose-free DMEM (Gibco) for 4 h before they were cultured in DMEM containing 4.5 g/L ^13^C-labeled glucose for 24 h. Cells were washed twice with cold PBS, and polar metabolites were extracted by 500 μL of ice-cold 80% methanol immediately. Samples were sonicated after repeated freezing and thawing. After centrifuging at 20,000 × *g* for 15 min, the supernatant was collected and dried. The pellets were dissolved in 50 μL 80% methanol for LC-MS detection. For LC/MS analysis, an ExionLC^TM^ UHPLC system combined with an AB SCIEX tripleTOF^TM^ 5600+ system was used. Samples were injected into a C18 column (2.1 × 100 mm, 3 μm, Guangzhou FLM Scientific Instrument Co., Ltd) with a flow rate of 0.3 mL/min. The mobile phase of LC-MS/MS was composed of 100% Milli-Q water (A) and 0.1% formic acid in acetonitrile (B) in negative ion mode. The mobile phase (A) was held at 10% for 5 min, increased to 90% in 7 min, and held for 2 min; then the mobile phase (A) was returned to the 10% A phase in 6 s and held for 3 min. Data were acquired and analyzed using Sciex PeakView 2.2 software.

### Xenograft model

Mouse experiments were conducted at the Experimental Animal Center of University of Science and Technology of China. A total of 3 × 10^6^ HepG2 cells transduced with the indicated lentiviruses were subcutaneously injected into the dorsal flanks of 4-week-old male nude mice (Nanjing GemPharmatech Co. Ltd.) (*n* = 6 for each group). Mice were used in the experiment at random. After injection, tumor volumes were measured every 6 days with a caliper and calculated using the equation: volume = length × width^2^ × 0.52. Twenty-four days after injection, the mice were sacrificed, and tumors were excised and weighed. During testing the tumors’ weight, the experimentalists were blinded to the information of tumor tissues. The RNA and protein extracts from the excised tumors were subjected to RT-PCR and western blot analyses, respectively.

### Ethics statement

All experiments with human tissue specimens were approved by the Ethics Committee of University of Science and Technology of China. All samples were collected with the informed consent of patients. The mouse experiments were approved by the Animal Research Ethics Committee of the University of Science and Technology of China and were performed in accordance with relevant guidelines and regulations.

### Reproducibility

Each experiment was repeated independently with similar results at least three times. The shown images are representative of three independent experiments.

### Statistical analysis

Statistical analysis was performed using Microsoft Excel software and GraphPad Prism to assess the differences between experimental groups. Statistical significance was analyzed by two-tailed Student’s t test, one-way ANOVA or two-way ANOVA with Tukey’s post-test and expressed as a *P* value. *P* < 0.05 was considered to be statistically significant. *, *P* < 0.05; **, *P* < 0.01; ***, *P* < 0.001; ns., no significance.

## Results

### Identification of circDDX21, a glucose deprivation-induced circular RNA implicated in glycolysis

**Fig. 1:**
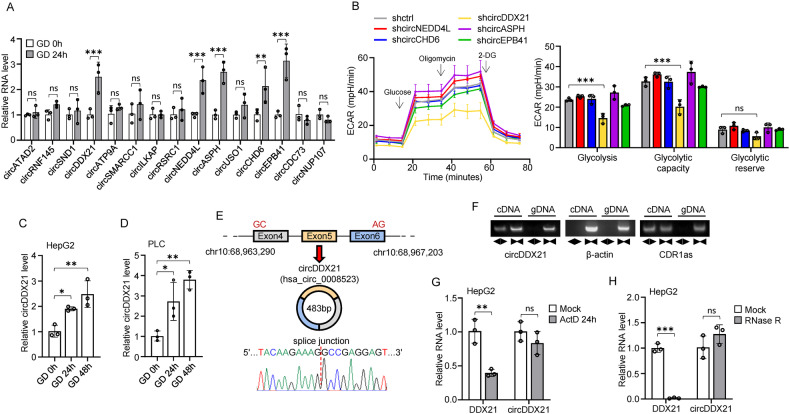
Glucose deprivation-induced circDDX21 promotes glycolysis. **A** Real-time RT-PCR analysis of the levels of the indicated circRNAs in HepG2 cells cultured in glucose-deprived (GD) medium for the indicated periods of time. Data shown are mean ± SD (n = 3), **p < 0.01, ***p < 0.001, ns., no significance. **B** ECAR was measured by Seahorse XF assay in HepG2 cells expressing shRNAs against the indicated circRNAs. The levels of glycolysis, glycolytic capacity, and glycolytic reserve were also calculated. Real-time RT-PCR analysis of circDDX21 levels in HepG2 (**C**) and PLC (**D**) cells cultured in glucose-deprived (GD) medium for the indicated periods of time. Data shown are mean ± SD (n = 3), *p < 0.05, **p < 0.01. **E** Sanger sequencing analysis of head-to-tail splicing junction in circDDX21. **F** cDNA and gDNA from HepG2 cells were used as templates to amplify circDDX21, β-actin, and CDR1as with divergent and convergent primers as indicated. **G** Real-time RT-PCR analysis of DDX21 and circDDX21 levels in HepG2 cells treated with or without actinomycin D (2 μg/ml) for 24 h. Data shown are mean ± SD (n = 3), **p < 0.01, ns., no significance. **H** Real-time RT-PCR analysis of DDX21 and circDDX21 levels in total RNA from HepG2 cells after treatment with or without RNase R (3 U/μg) for 30 min. Data shown are mean ± SD (n = 3), ***p < 0.001, ns., no significance.

To identify novel circular RNAs involved in the regulation of glucose metabolism, we conducted RNA sequencing analysis. We observed the induction of twenty-eight circRNAs upon glucose deprivation in hepatocellular carcinoma (HCC) HepG2 cells (fold change > 2.0; p < 0.05) (Supplementary Table 1). Among these circRNAs, we selected the top 15 significantly upregulated circular RNAs in response to glucose deprivation for further validation using real-time RT-PCR (Fig. 1A, Supplementary Table 1). Five circRNAs, including circDDX21, circNEDD4L, circASPH, circCHD6, and circEPB41, were confirmed to be upregulated in HepG2 cells upon glucose deprivation (Fig. 1A). To investigate the potential impact of these five circRNAs on glycolysis, we conducted Seahorse assays to measure the extracellular acidification rate (ECAR). Intriguingly, knockdown of circDDX21, but not the other circRNAs, led to a dramatic decrease in the glycolytic rate in HepG2 cells (Fig. 1B, Supplementary Fig. 1A), indicating the specific regulatory effect of circDDX21 on glycolysis. Subsequent real-time RT-PCR analysis showed that both the relative expression and copy number of circDDX21 in HepG2 and PLC cells were greatly increased upon glucose deprivation (Fig. 1C, D, Supplementary Fig. 1B, C), reinforcing the inducing effect of glucose deprivation on circDDX21 expression.

According to circBase, circDDX21 (hsa_circ_0008523) is a 483-nt circRNA generated by back-splicing of exons 4, 5, and 6 of the *DDX21* gene located on human chromosome 10. Sanger sequencing validated that the back-splicing junction region matched the reported sequence (Fig. 1E). The back-splicing of endogenous circDDX21 was also confirmed by PCR using convergent and divergent primers, with β-actin and CDR1as serving as controls for linear and circular RNAs, respectively. As expected, the convergent primers amplified PCR products from both complementary DNA (cDNA) and genomic DNA (gDNA) templates for circDDX21, β-actin, and CDR1as (Fig. 1F). However, when cDNA was used as a template, the divergent primers amplified a PCR product for circDDX21 and CDR1as but not for β-actin (Fig. 1F). Actinomycin D chase assays revealed that circDDX21 exhibited an extended half-life compared to linear DDX21 mRNA (Fig. 1G, Supplementary Fig. 1D). Unlike DDX21 mRNA, which was susceptible to degradation by the exonuclease RNase R, circDDX21 remained stable following RNase R treatment (Fig. 1H, Supplementary Fig. 1E). Fluorescence in situ hybridization (FISH) and cellular fractionation experiments revealed that circDDX21 was predominantly localized in the cytoplasm of HepG2 cells (Supplementary Fig. 1F, G). To exclude the protein-coding possibility of circDDX21, we cloned the predicted internal ribosome entry site (IRES) (154–328 bp) in circDDX21 into a split GFP reporter construct (Supplementary Fig. 1H). The results showed that this predicted IRES was incapable of mediating GFP translation (Supplementary Fig. 1H). Taken together, these data establish circDDX21 as a glucose deprivation-induced circRNA implicated in glycolysis.

### c-Myc mediates circDDX21 induction in response to glucose deprivation

**Fig. 2:**
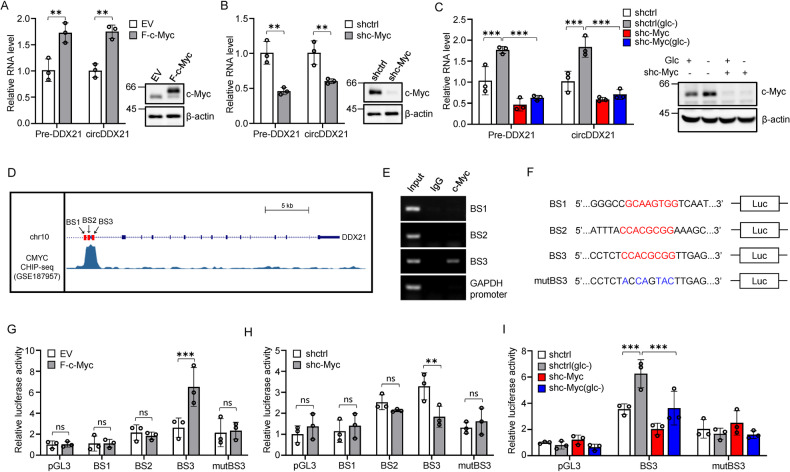
c-Myc drives circDDX21 production in response to glucose deprivation. **A** Real-time RT-PCR analysis of pre-DDX21 and circDDX21 levels in HepG2 cells with or without ectopic expression of c-Myc. Data shown are mean ± SD (n = 3), **p < 0.01. The successful overexpression of c-Myc was evaluated by western blotting. **B** Real-time RT-PCR analysis of pre-DDX21 and circDDX21 levels in HepG2 cells expressing control shRNA or c-Myc shRNA. Data shown are mean ± SD (n = 3), **p < 0.01. The knockdown efficiency of c-Myc was evaluated by western blotting. **C** HepG2 cells expressing control shRNA or c-Myc shRNA were cultured in normal or glucose-deprived (GD) medium for 24 h, followed by real-time RT-PCR analysis. Data shown are mean ± SD (n = 3), ***p < 0.001. Cell lysates were also analyzed by western blotting. **D** UCSC Genome Browser illustration of Encode ChIP-seq data for c-Myc in HepG2 cells. BS1, BS2, and BS3 represent three putative c-Myc binding sites predicted by the JASPAR database. **E** ChIP assay was conducted in HepG2 cells using an anti-c-Myc antibody or a control IgG. **F** Shown are the pGL3-based wild-type and mutant reporter plasmids used for luciferase assay. **G** HepG2 cells expressing empty vector (EV) or Flag-c-Myc were co-transfected with the indicated pGL3-based reporter constructs plus Renilla luciferase plasmid. Twenty-four hours after transfection, reporter activity was measured. Data shown are mean ± SD (n = 3). ***p < 0.001, ns., no significance. **H** HepG2 cells expressing control shRNA or c-Myc shRNA were co-transfected with the indicated pGL3-based reporter constructs plus Renilla luciferase plasmid. Twenty-four hours after transfection, reporter activity was measured. Data shown are mean ± SD (n = 3). **p < 0.01, ns., no significance. **I** HepG2 cells expressing control shRNA or c-Myc shRNA were co-transfected with the indicated pGL3-based reporter constructs plus Renilla luciferase plasmid. Twenty-four hours after transfection, cells were cultured in normal or glucose-deprived medium for 24 h. Reporter activity was then measured. Data shown are mean ± SD (n = 3). ***p < 0.001.

We next explored how circDDX21 is upregulated by glucose deprivation. Treatment of both HepG2 and PLC cells with glucose deprivation not only induced the expression of circDDX21 but also elevated pre-DDX21 mRNA levels (Supplementary Fig. 2A, B), indicating that circDDX21 expression induced by glucose deprivation occurs at the transcriptional level. Analysis of the promoter and intronic regions of the DDX21 gene using the JASPAR database revealed potential binding motifs for three transcription factors: YY1, c-Myc, and NRF1, known to be functionally associated with metabolic regulation [45–47] (Supplementary Fig. 2C). Knockdown of c-Myc, but not others, significantly decreased circDDX21 expression under glucose deprivation in HepG2 cells (Supplementary Fig. 2D, E), implying that c-Myc is responsible for up-the regulation of circDDX21. In support of this, ectopic expression of c-Myc increased, whereas knockdown of c-Myc decreased, the levels of both pre-DDX21 and circDDX21 in HepG2 and PLC cells (Fig. 2A, B, Supplementary Fig. 2F, G). Moreover, knockdown of c-Myc completely reversed the inducing effect of glucose deprivation on pre-DDX21 and circDDX21 expression in HepG2 cells (Fig. 2C).

To further investigate whether c-Myc transcriptionally upregulates circDDX21 expression, we performed chromatin immunoprecipitation (ChIP) and luciferase reporter assays. Analysis of both the JASPAR and ENCODE databases revealed three putative c-Myc binding sites (BS1, BS2, and BS3) located around the transcription start site of the DDX21 gene (Fig. 2D). The ChIP assay verified the association of c-Myc with the chromatin fragment comprising the BS3 site (Fig. 2E). To explore whether the BS3 site is responsible for c-Myc-dependent transcriptional activity, we generated a series of pGL3-based luciferase reporter constructs (Fig. 2F). The luciferase reporter assay showed that the luciferase activity from the reporter construct containing the wild-type BS3 site, but not the mutant BS3 site, was induced by c-Myc overexpression and reduced by c-Myc knockdown (Fig. 2G, H). In addition, neither overexpression nor knockdown of c-Myc affected the luciferase activity from the reporter construct containing the BS1 or BS2 site (Fig. 2G, H). Moreover, the enhancing effect of glucose deprivation on the luciferase activity from the reporter construct containing the wild-type BS3 site was substantially diminished by c-Myc knockdown (Fig. 2I). Collectively, these findings suggest that c-Myc drives the production of circDDX21 in response to glucose deprivation.

### circDDX21 promotes glycolysis by increasing PGAM1 expression

**Fig. 3:**
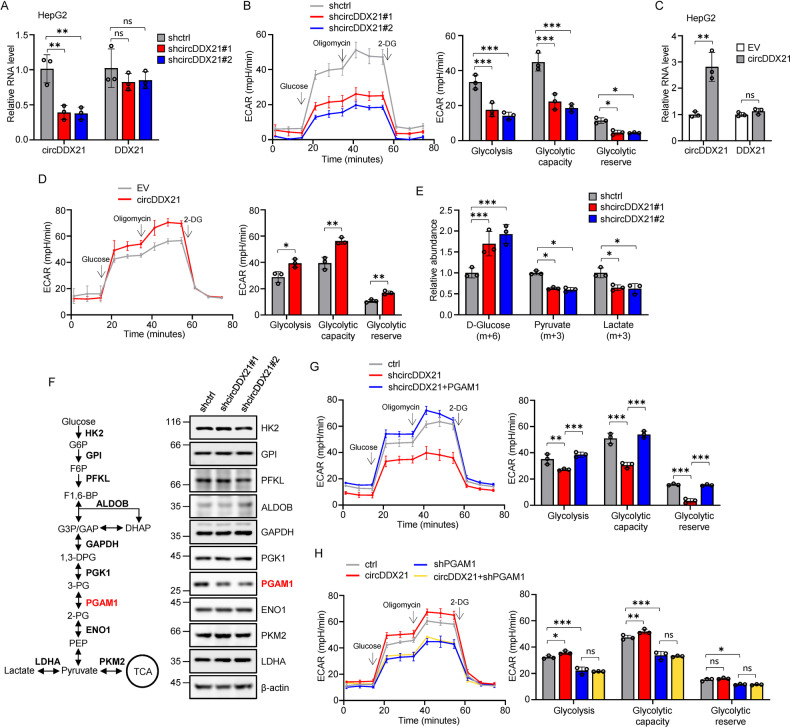
circDDX21 promotes glycolysis by increasing PGAM1 expression. **A** Real-time RT-PCR analysis of circDDX21 and DDX21 levels in HepG2 cells transduced with lentiviruses expressing control shRNA, circDDX21 shRNA#1, or circDDX21 shRNA#2. Data shown are mean ± SD (n = 3), **p < 0.01, ns., no significance. **B** ECAR was measured by Seahorse XF assay in HepG2 cells expressing control shRNA, circDDX21 shRNA#1, or circDDX21 shRNA#2. The levels of glycolysis, glycolytic capacity, and glycolytic reserve were also calculated. Data shown are mean ± SD (n = 3), *p < 0.05, ***p < 0.001. **C** Real-time RT-PCR analysis of circDDX21 and DDX21 levels in HepG2 cells transduced with lentiviruses expressing empty vector (EV) or circDDX21. Data shown are mean ± SD (n = 3), **p < 0.01, ns., no significance. **D** ECAR was measured by Seahorse XF assay in HepG2 cells expressing empty vector (EV) or circDDX21. The levels of glycolysis, glycolytic capacity, and glycolytic reserve were also calculated. Data shown are mean ± SD (n = 3), *p < 0.05, **p < 0.01. **E** The glycolytic tracing assay was performed with ^13^C-labeled glucose in HepG2 cells expressing control shRNA, circDDX21 shRNA#1, or circDDX21 shRNA#2. The relative abundance of the ^13^C-labeled glycolytic metabolites D-glucose, pyruvate, and lactate was calculated. Data shown are mean ± SD (n = 3), *p < 0.05, ***p < 0.001. **F** Lysates from HepG2 cells expressing control shRNA, circDDX21 shRNA#1, or circDDX21 shRNA#2 were analyzed by western blotting to examine the protein levels of the indicated enzymes involved in the glycolysis pathway. **G** HepG2 cells were infected with lentiviruses expressing control, circDDX21 shRNA, or circDDX21 shRNA plus PGAM1. Forty-eight hours after infection, the ECAR was measured by a Seahorse XF assay. The levels of glycolysis, glycolytic capacity, and glycolytic reserve were also calculated. Data shown are mean ± SD (n = 3), **p < 0.01, ***p < 0.001. **H** HepG2 cells were infected with lentiviruses expressing control, circDDX21, PGAM1 shRNA, or both circDDX21 and PGAM1 shRNA. Forty-eight hours after infection, the ECAR was measured by a Seahorse XF assay. The levels of glycolysis, glycolytic capacity, and glycolytic reserve were also calculated. Data shown are mean ± SD (n = 3), *p < 0.05, **p < 0.01, ***p < 0.001, ns., no significance.

The above-mentioned inhibitory effect of circDDX21 knockdown on the glycolytic rate (Fig. 1B) prompted us to further investigate the role of circDDX21 in glycolysis. Two independent shRNA-mediated knockdown of circDDX21 consistently resulted in a dramatic decrease in the glycolytic rate in HepG2 cells (Fig. 3A, B). Conversely, ectopic expression of circDDX21 markedly increased the glycolytic rate in HepG2 cells (Fig. 3C, D), demonstrating that circDDX21 promotes glycolysis. In support of this, the glycolytic tracing assay with ^13^C-labeled glucose showed that the cellular levels of ^13^C-labeled glucose were significantly elevated, whereas the cellular levels of ^13^C-labeled pyruvate and lactate were greatly reduced upon circDDX21 knockdown in HepG2 cells (Fig. 3E).

To explore the molecular mechanisms by which circDDX21 promotes glycolysis, we first examined the effect of circDDX21 knockdown on the protein levels of all glycolytic enzymes. Intriguingly, knockdown of circDDX21 specifically decreased the protein levels of PGAM1 without affecting the protein expression of other examined glycolytic enzymes in HepG2 cells (Fig. 3F). In contrast, overexpression of circDDX21 strongly increased the protein levels of PGAM1 in HepG2 cells (Supplementary Fig. 3A). This promoting effect of circDDX21 on PGAM1 protein expression was also validated in both circDDX21-overexpressing and knockdown PLC cells (Supplementary Fig. 3B-E). Of note, neither knockdown nor overexpression of circDDX21 affected DDX21 expression in both HepG2 and PLC cells (Fig. 3A, C, Supplementary Fig. 3C, E). Moreover, knockdown or overexpression of circDDX21 showed no obvious effects on PGAM1 mRNA and protein levels (Supplementary Fig. 3F, G). These data indicate that cirDDX21 increases PGAM1 expression independent of DDX21.

To further determine whether circDDX21 promotes glycolysis through the regulation of PGAM1, we performed rescue experiments. Knockdown of circDDX21 consistently led to a reduction in the glycolytic rate in HepG2 cells (Fig. 3G). However, this suppressive effect caused by circDDX21 knockdown could be reversed by ectopic expression of PGAM1 (Fig. 3G). In addition, circDDX21 overexpression was able to increase the glycolytic rate in control HepG2 cells but not in PGAM1 knockdown HepG2 cells (Fig. 3H). Together, these data suggest that circDDX21 promotes glycolysis by increasing PGAM1 expression.

### circDDX21 coordinates with PABPC1 to increase PGAM1 mRNA stability

**Fig. 4:**
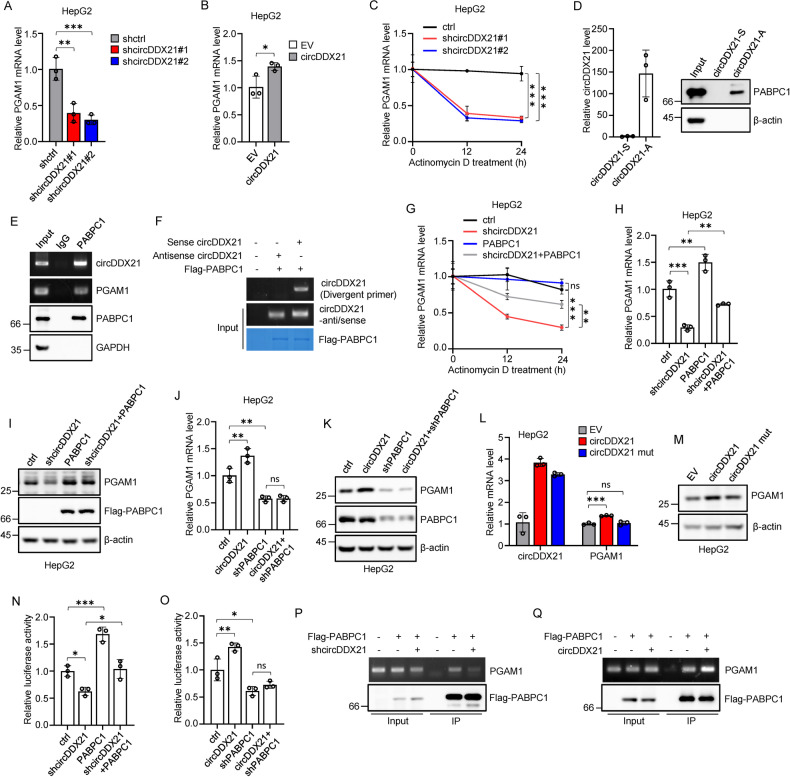
circDDX21 cooperates with PABPC1 to increase PGAM1 mRNA stability. **A** Real-time RT-PCR analysis of PGAM1 mRNA levels in HepG2 cells transduced with lentiviruses control shRNA, circDDX21 shRNA#1, or circDDX21 shRNA#2. Data shown are mean ± SD (n = 3), **p < 0.01, ***p < 0.001. **B** Real-time RT-PCR analysis of PGAM1 mRNA levels in HepG2 cells transduced with lentiviruses empty vector (EV) or circDDX21. Data shown are mean ± SD (n = 3), *p < 0.05. **C** HepG2 cells transduced with lentiviruses control shRNA, circDDX21 shRNA#1, or circDDX21 shRNA#2 were incubated with actinomycin D (2 μg/mL) for the indicated periods of time, followed by real-time RT-PCR analysis to examine PGAM1 mRNA stability. Data shown are mean ± SD (n = 3), ***p < 0.001. **D** Lysates from HepG2 cells were subjected to a biotin pull-down assay using sense or antisense biotin-labeled DNA oligomers corresponding to circDDX21. The pull-down complexes were analyzed by real-time RT-PCR and western blotting. **E** Lysates from HepG2 cells were subjected to RNA immunoprecipitation using an anti-PABPC1 antibody or a control IgG. The input and immunoprecipitates were analyzed by RT-PCR and western blotting. **F** Purified recombinant Flag-PABPC1 bound with anti-Flag M2 beads was incubated with in vitro-synthesized circDDX21 or its antisense RNA. The bead-bound RNAs were then analyzed by RT-PCR. **G** HepG2 cells transduced with lentiviruses expressing control, circDDX21 shRNA, PABPC1, or both circDDX21 shRNA and PABPC1 were incubated with actinomycin D (2 μg/mL) for the indicated periods of time, followed by real-time RT-PCR analysis to examine PGAM1 mRNA stability. Data shown are mean ± SD (n = 3), **p < 0.01, ***p < 0.001, ns., no significance. **H** Real-time RT-PCR analysis of PGAM1 mRNA levels in HepG2 cells transduced with lentiviruses expressing control, circDDX21 shRNA, PABPC1, or both circDDX21 shRNA and PABPC1. Data shown are mean ± SD (n = 3), **p < 0.01, ***p < 0.001. **I** Western blot analysis of PGAM1 protein levels in HepG2 cells transduced with lentiviruses expressing control, circDDX21 shRNA, Flag-PABPC1, or both circDDX21 shRNA and Flag-PABPC1. **J** Real-time RT-PCR analysis of PGAM1 mRNA levels in HepG2 cells transduced with lentiviruses expressing control, circDDX21, PABPC1 shRNA, or both circDDX21 and PABPC1 shRNA. Data shown are mean ± SD (n = 3), **p < 0.01, ns., no significance. **K** Western blot analysis of PGAM1 protein levels in HepG2 cells transduced with lentiviruses expressing control, circDDX21, PABPC1 shRNA, or both circDDX21 and PABPC1 shRNA. **L** Real-time RT-PCR analysis of PGAM1 mRNA levels in HepG2 cells with stable expression of circDDX21 or the circDDX21 mutant (ΔPABPC1 BS). Data shown are mean ± SD (n = 3). ***p < 0.001; ns., no significance. **M** Western blot analysis of PGAM1 protein levels in HepG2 cells with stable expression of circDDX21 or the circDDX21 mutant (ΔPABPC1 BS). **N** HepG2 cells expressing control, circDDX21 shRNA, Flag-PABPC1, or both circDDX21 shRNA and Flag-PABPC1 were transfected with psiCHECK2-PGAM1-3′UTR. Twenty-four hours later, reporter activity was measured. Data shown are mean ± SD (n = 3). *p < 0.05, ***p < 0.001. **O** HepG2 cells expressing control, circDDX21, PABPC1 shRNA, or both circDDX21 and PABPC1 shRNA were transfected with psiCHECK2-PGAM1-3′UTR. Twenty-four hours later, reporter activity was measured. Data shown are mean ± SD (n = 3). *p < 0.05, **p < 0.01, ns, no significance. **P** HepG2 cells transduced with lentiviruses expressing circDDX21 shRNA and Flag-PABPC1 in the indicated combination, followed by an RNA immunoprecipitation assay using anti-Flag antibody. The input and immunoprecipitates were analyzed by RT-PCR and western blotting. **Q** HepG2 cells transduced with lentiviruses expressing circDDX21 and Flag-PABPC1 in the indicated combination, followed by an RNA immunoprecipitation assay using anti-Flag antibody. The input and immunoprecipitates were analyzed by RT-PCR and western blotting.

We next investigated how circDDX21 increases PGAM1 expression. Real-time RT-PCR analysis showed that knockdown of circDDX21 dramatically decreased PGAM1 mRNA levels, while overexpression of circDDX21 exhibited the opposite effect in both HepG2 and PLC cells (Fig. 4A, B, Supplementary Fig. 4A, B). Given the predominant cytoplasmic localization of circDDX21 (Supplementary Fig. 1F, G), we hypothesized that circDDX21 might regulate PGAM1 mRNA stability. To test this hypothesis, HepG2 and PLC cells with circDDX21 knockdown were treated with actinomycin D to measure the half-life of PGAM1 mRNA. Knockdown of circDDX21 in these cells strongly decreased the half-life of PGAM1 mRNA (Fig. 4C, Supplementary Fig. 4C), indicating the stabilizing effect of circDDX21 on PGAM1 mRNA. To explore how circDDX21 promotes PGAM1 mRNA stability, we sought to identify circDDX21-interacting proteins. Proteins specifically pulled down by antisense DNA oligomers against circDDX21 were separated by SDS-PAGE and analyzed by mass spectrometry (Supplementary Fig. 4D). Among the top ten identified potential circDDX21-binding protein candidates, three candidates, ENO1, UPF1, and PABPC1, with primary cytoplasmic localization, were selected for further functional verification (Supplementary Fig. 4E). Knockdown of either ENO1 or UPF1 did not show any noticeable effect on PGAM1 mRNA levels, while knockdown of PABPC1 resulted in decreased PGAM1 mRNA and protein levels (Supplementary Fig. 4F, G). Moreover, similar to the inhibitory effect observed upon circDDX21 knockdown, knockdown of PABPC1 also accelerated the decay of PGAM1 mRNA (Supplementary Fig. 4H). Based on these findings, PABPC1 was chosen for further investigation.

To verify the interaction between PABPC1 and circDDX21, an RNA pull-down assay was performed. The results showed that PABPC1 was indeed specifically pulled down by antisense, but not sense, DNA oligomers corresponding to circDDX21 (Fig. 4D). The PABPC1-circDDX21 interaction was also validated by an RNA immunoprecipitation assay (Fig. 4E). Notably, PABPC1 was also found to interact with PGAM1 mRNA (Fig. 4E). Additionally, an in vitro binding assay revealed that PABPC1 directly interacted with circDDX21 but not its antisense RNA (Fig. 4F, Supplementary Fig. 4I). To identify the PABPC1-binding region in circDDX21, RBPmap was used for prediction [48]. Subsequent in vitro binding assay showed that unlike wild-type circDDX21, mutant circDDX21 (ΔPABPC1 BS) with mutation of the predicted PABPC1-binding site did not exhibit obvious PABPC1-binding ability (Supplementary Fig. 4J, K). These data support that PABPC1 is a binding partner for circDDX21.

We next evaluated whether circDDX21 positively regulates PGAM1 mRNA stability via PABPC1. The results showed that the decreased PGAM1 mRNA stability caused by circDDX21 knockdown could be significantly restored by overexpression of PABPC1. (Fig. 4G). In accordance, the decreased mRNA and protein levels of PGAM1 resulting from circDDX21 knockdown could be markedly rescued by ectopically expressed PABPC1 (Fig. 4H, I, Supplementary Fig. 4L, M). In addition, circDDX21 overexpression was shown to increase the mRNA and protein levels of PGAM1 in control cells, but not in PABPC1 knockdown cells (Fig. 4J, K, Supplementary Fig. 4N, O). Moreover, compared to wild-type circDDX21, mutant circDDX21 (ΔPABPC1 BS) without PABPC1-binding ability did not exhibit noticeable effects on PGAM1 mRNA and protein levels (Fig. 4L, M). These data indicate that the regulatory effect of circDDX21 on PGAM1 expression is dependent on PABPC1. Considering the 3’-UTR is critical for controlling mRNA stability, we cloned the PGAM1 3’-UTR into the psiCHECK2 luciferase reporter construct (Supplementary Fig. 4P). As expected, the reporter activity from the psiCHECK2-PGAM1 3’-UTR was reduced by circDDX21 knockdown and increased by circDDX21 overexpression (Fig. 4N, O). The inhibitory effect of circDDX21 knockdown on PGAM1 3’-UTR reporter activity could be reversed by PABPC1 overexpression (Fig. 4N). In addition, circDDX21 failed to increase PGAM1 3’-UTR reporter activity when PABPC1 was knocked down (Fig. 4O). These findings indicate that the stabilizing effect of circDDX21 on PGAM1 mRNA depends on PABPC1. These results also raised the question of whether circDDX21 could facilitate the binding of PABPC1 to PGAM1 mRNA, thereby enhancing PGAM1 mRNA stability. To address this, an RNA immunoprecipitation assay was performed. The results showed that knockdown of circDDX21 attenuated, whereas overexpression of circDDX21 increased, the binding of PABPC1 to PGAM1 mRNA (Fig. 4P, Q). Taken together, these data suggest that circDDX21 cooperates with PABPC1 to promote PGAM1 mRNA stabilization.

### circDDX21 increases the binding of PABPC1 to PGAM1 mRNA by inhibiting MKRN3-mediated PABPC1 ubiquitination

**Fig. 5:**
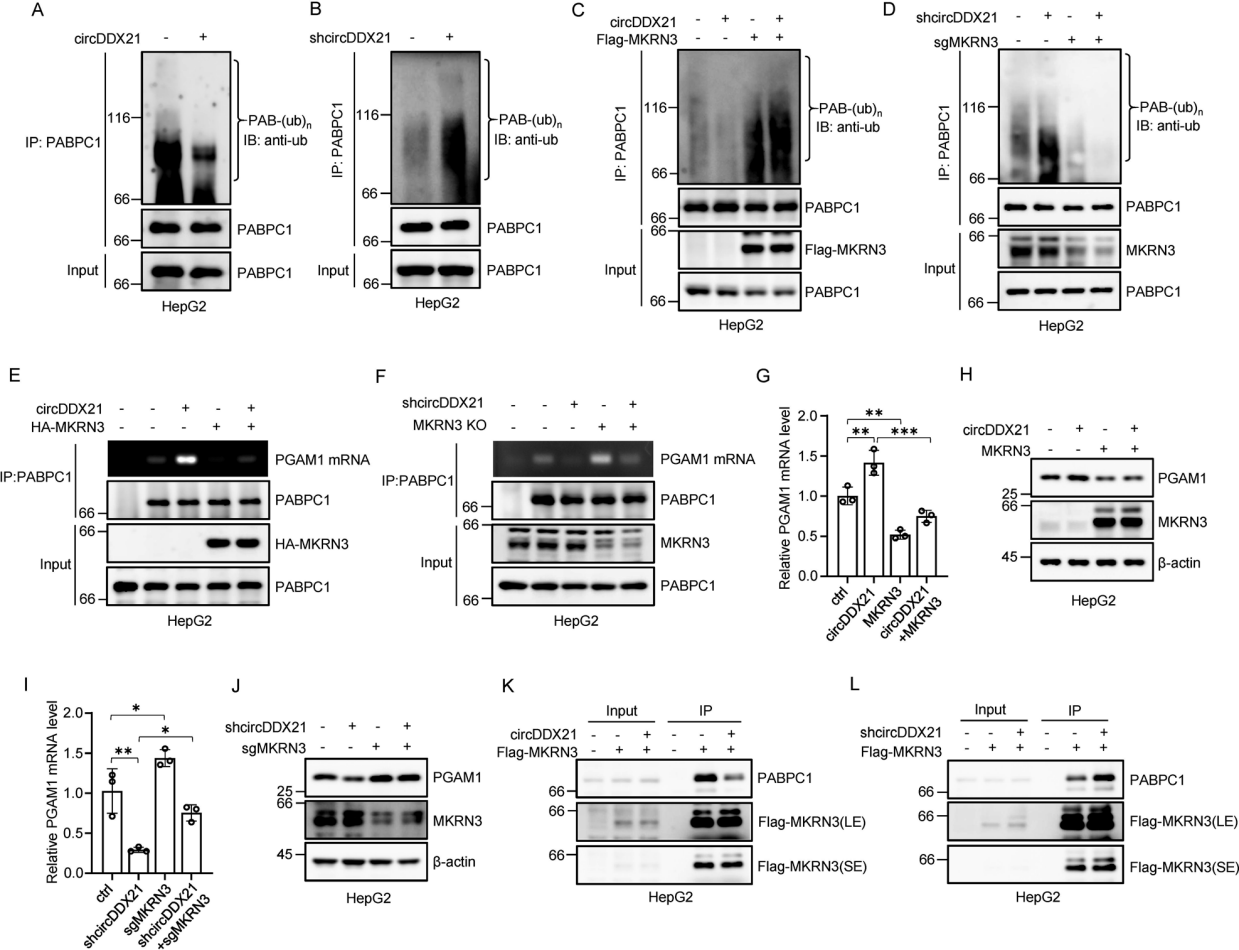
circDDX21 increases the binding of PABPC1 to PGAM1 mRNA by inhibiting MKRN3-mediated PABPC1 ubiquitination. **A** Lysates from HepG2 cells transduced with lentiviruses expressing empty vector (EV) or circDDX21 were subjected to an in vivo ubiquitination assay using anti-PABPC1 antibody. **B** Lysates from HepG2 cells transduced with lentiviruses expressing control shRNA or circDDX21 shRNA were subjected to an in vivo ubiquitination assay using an anti-PABPC1 antibody. **C** HepG2 cells were infected with lentiviruses expressing circDDX21 and Flag-MKRN3 in the indicated combination, followed by an in vivo ubiquitination assay using an anti-PABPC1 antibody. **D** HepG2 cells were infected with lentiviruses expressing circDDX21 shRNA and MKRN3 sgRNA in the indicated combination, followed by an in vivo ubiquitination assay using an anti-PABPC1 antibody. **E** HepG2 cells were infected with lentiviruses expressing circDDX21 and HA-MKRN3 in the indicated combination, followed by an RNA immunoprecipitation assay using an anti-PABPC1 antibody. **F** HepG2 cells were infected with lentiviruses expressing circDDX21 shRNA and MKRN3 sgRNA in the indicated combination, followed by an RNA immunoprecipitation assay using an anti-PABPC1 antibody. **G** HepG2 cells were infected with lentiviruses expressing circDDX21 and Flag-MKRN3 in the indicated combination, followed by real-time RT-PCR analysis of PGAM1 mRNA levels. Data shown are mean ± SD (n = 3). **p < 0.01, ***p < 0.001. **H** HepG2 cells were infected with lentiviruses expressing circDDX21 and Flag-MKRN3 in the indicated combination, followed by western blot analysis of PGAM1 protein levels. **I** HepG2 cells were infected with lentiviruses expressing circDDX21 shRNA and MKRN3 sgRNA in the indicated combination, followed by real-time RT-PCR analysis of PGAM1 mRNA levels. Data shown are mean ± SD (n = 3). *p < 0.05, **p < 0.01. **J** HepG2 cells were infected with lentiviruses expressing circDDX21 shRNA and MKRN3 sgRNA in the indicated combination, followed by western blot analysis of PGAM1 protein levels. **K** HepG2 cells were infected with lentiviruses expressing circDDX21 and Flag-MKRN3 in the indicated combination, followed by an immunoprecipitation assay using anti-Flag antibody. **L** HepG2 cells were infected with lentiviruses expressing circDDX21 shRNA and Flag-MKRN3 in the indicated combination, followed by an immunoprecipitation assay using anti-Flag antibody.

We next sought to investigate how circDDX21 enhances the binding of PABPC1 to PGAM1 mRNA. It has been previously shown that MKRN3-mediated ubiquitination attenuates the binding of PABPC1 to its target mRNA [49, 50]. We therefore asked whether circDDX21 could regulate PABPC1 ubiquitination, thereby affecting the interaction between PABPC1 and PGAM1 mRNA. By performing an in vivo ubiquitination assay, we showed that overexpression of wild-type circDDX21, but not PABPC1 binding-defective mutant of circDDX21 (ΔPABPC1 BS), greatly decreased PABPC1 ubiquitination (Fig. 5A, Supplementary Fig. 5A). Conversely, knockdown of circDDX21 strongly increased PABPC1 ubiquitination (Fig. 5B). To determine whether the inhibitory effect of circDDX21 on PABPC1 ubiquitination is dependent on MKRN3, we performed rescue experiments. In agreement with previous reports [49, 50], ubiquitination of endogenous PABPC1 was induced by MKRN3 overexpression and reduced by MKRN3 knockdown (Fig. 5C, D), reinforcing the importance of MKRN3 in controlling PABPC1 ubiquitination. Moreover, the circDDX21-mediated suppression of PABPC1 ubiquitination could be reversed by ectopic expression of MKRN3 (Fig. 5C), while knockdown of MKRN3 completely abolished the enhanced PABPC1 ubiquitination caused by circDDX21 knockdown (Fig. 5D), indicating that circDDX21 suppresses MKRN3-mediated PABPC1 ubiquitination.

Consistent with the role of MKRN3 in circDDX21-regulated PABPC1 ubiquitination, the enhancing effect of circDDX21 on the PABPC1-PGAM1 mRNA interaction was strongly minimized by MKRN3 overexpression (Fig. 5E). In addition, the decreased PABPC1-PGAM1 mRNA interaction resulting from circDDX21 knockdown could be substantially rescued by concurrent knockdown of MKRN3 (Fig. 5F). Collectively, these data suggest that circDDX21 enhances the binding of PABPC1 to PAGM1 mRNA by suppressing MKRN3-mediated PABPC1 ubiquitination. In accordance, circDDX21 overexpression was shown to induce the mRNA and protein expression of PGAM1 in control cells, but not in MKRN3-overexpressing cells (Fig. 5G, H). Moreover, the reduction in mRNA and protein levels of PGAM1 caused by circDDX21 knockdown could be greatly recovered by concurrent knockdown of MKRN3 (Fig. 5I, J).

We next explored how circDDX21 suppresses MKRN3-mediated PABPC1 ubiquitination. The immunoprecipitation assay revealed that the interaction between MKRN3 and PABPC1 was compromised by circDDX21 overexpression (Fig. 5K). In contrast, circDDX21 knockdown enhanced the MKRN3-PABPC1 interaction (Fig. 5L). By performing mapping experiments, circDDX21 was shown to associate with both the central region (aa 181-380) and the C-terminal region (aa 381-636) of PABPC1 (Supplementary Fig. 5B, C). This C-terminal region (aa 381-636) of PABPC1 has been previously reported to mediate the interaction with MKRN3 [49]. Therefore, our data imply that circDDX21 competes with MKRN3 for binding to the C-terminal region (aa 381-636) of PABPC1, thereby disrupting the MKRN3-PABPC1 association and suppressing MKRN3-mediated PABPC1 ubiquitination.

### Biological implication of circDDX21 in hepatocellular carcinogenesis

**Fig. 6:**
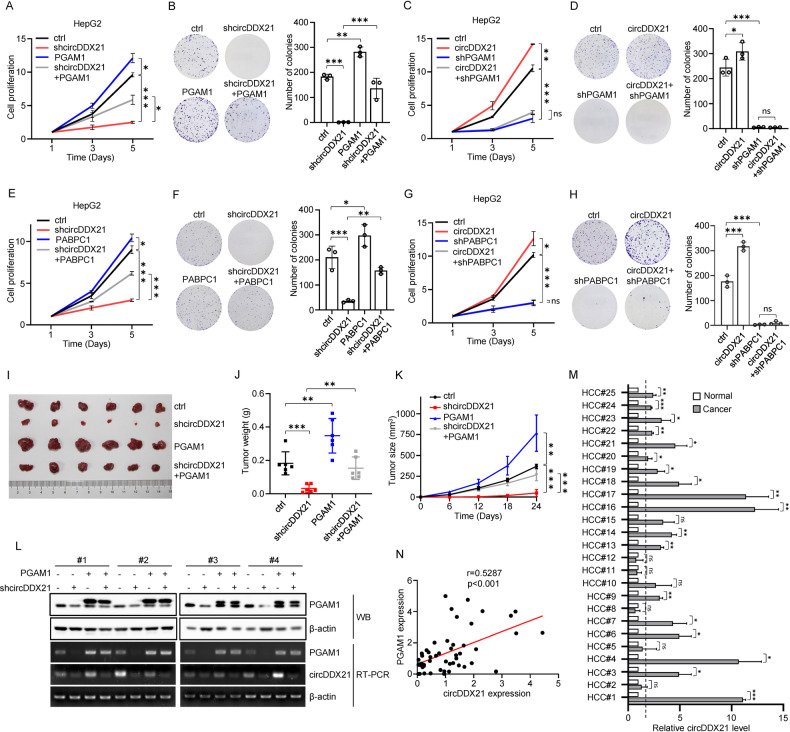
circDDX21 functions as an oncogenic circRNA to promote hepatocellular carcinogenesis. **A** Growth curves of HepG2 cells expressing control, circDDX21 shRNA, PGAM1, or both circDDX21 shRNA and PGAM1. Data shown are mean ± SD (n = 3). *p < 0.05, ***p < 0.001. **B** Colonies of HepG2 cells expressing control, circDDX21 shRNA, PGAM1, or both circDDX21 shRNA and PGAM1 were stained with crystal violet after 14 days of incubation. Data shown are mean ± SD (n = 3). **p < 0.01, ***p < 0.001. **C** Growth curves of HepG2 cells expressing control, circDDX21, PGAM1 shRNA, or both circDDX21 and PGAM1 shRNA. Data shown are mean ± SD (n = 3). **p < 0.01, ***p < 0.001, ns, no significance. **D** Colonies of HepG2 cells expressing control, circDDX21, PGAM1 shRNA, or both circDDX21 and PGAM1 shRNA were stained with crystal violet after 14 days of incubation. Data shown are mean ± SD (n = 3). *p < 0.05, ***p < 0.001, ns, no significance. **E** Growth curves of HepG2 cells expressing control, circDDX21 shRNA, PABPC1, or both circDDX21 shRNA and PABPC1. Data shown are mean ± SD (n = 3). *p < 0.05, ***p < 0.001. **F** Colonies of HepG2 cells expressing control, circDDX21 shRNA, PABPC1, or both circDDX21 shRNA and PABPC1 were stained with crystal violet after 14 days of incubation. Data shown are mean ± SD (n = 3). *p < 0.05, **p < 0.01, ***p < 0.001. **G** Growth curves of HepG2 cells expressing control, circDDX21, PABPC1 shRNA, or both circDDX21 and PABPC1 shRNA. Data shown are mean ± SD (n = 3). *p < 0.05, ***p < 0.001, ns, no significance. **H** Colonies of HepG2 cells expressing control, circDDX21, PABPC1 shRNA, or both circDDX21 and PABPC1 shRNA were stained with crystal violet after 14 days of incubation. Data shown are mean ± SD (n = 3). ***p < 0.001, ns, no significance. A total of 3 × 10^6^ HepG2 cells expressing control, circDDX21 shRNA, PGAM1, or both circDDX21 shRNA and PGAM1 were individually injected into nude mice (n = 6 for each group). **I** Xenograft tumors were taken 24 days after injection. **J** Excised tumors were weighed. **p < 0.01, ***p < 0.001. **K** Tumor sizes were measured at the indicated time points. ***p < 0.001. **L** RNA and protein extracts from the excised xenografts were analyzed by RT-PCR and western blotting, respectively. **M** Real-time RT-CR analysis of circDDX21 levels among 25 pairs of matched hepatocellular carcinoma and adjacent normal tissue. Data shown are mean ± SD (n = 3). *p < 0.05, **p < 0.01, ***p < 0.001, ns., no significance. The dotted line indicates a fold change of 2. **N** Correlation analyses conducted between circDDX21 and PGAM1 mRNA expression in hepatocellular carcinoma samples (n = 51).

Given the promoting effect of circDDX21 on glycolysis in hepatocellular carcinoma cells, as demonstrated above, we asked whether circDDX21 could facilitate hepatocellular carcinogenesis. We first examined the effect of circDDX21 on the proliferation of hepatocellular carcinoma HepG2 cells. Knockdown of circDDX21 in HepG2 cells resulted in a dramatic decrease in proliferation and colony numbers (Fig. 6A, B). Conversely, overexpression of circDDX21 led to a strong increase in cell proliferation and colony formation (Fig. 6C, D). The inhibitory effects of circDDX21 knockdown on cell proliferation and colony formation were markedly reversed by ectopically expressed PGAM1 (Fig. 6A, B). In addition, circDDX21 no longer increased cell proliferation and colony formation when PGAM1 was knocked down (Fig. 6C, D). These data suggest that circDDX21 promotes hepatocellular carcinoma cell proliferation via PGAM1.

We next evaluated the functional role of PABPC1 in circDDX21-promoted cell proliferation. Ectopic expression of PABPC1 greatly rescued circDDX21 knockdown-induced decreases in cell proliferation and colony formation (Fig. 6E, F). In addition, circDDX21 overexpression consistently increased cell proliferation and colony formation in control cells, but not in PABPC1 knockdown cells (Fig. 6G, H), indicating that PABPC1 plays an important role in mediating the promoting effect of circDDX21 on cell proliferation. To determine whether circDDX21 promotes cell proliferation via the PABPC1-PGAM1 axis, we utilized PABPC1 binding-defective mutant of circDDX21 (ΔPABPC1 BS), which lost the ability to induce PGAM1 expression (Fig. 4L, M, Supplementary Fig. 4J, K). Unlike wild-type circDDX21, this mutant circDDX21 (ΔPABPC1 BS) failed to promote cell proliferation and colony formation (Supplementary Fig. 6A, B). In accordance, mutant circDDX21 (ΔPABPC1 BS) exhibited no obvious effect on the glycolytic rate compared to wild-type circDDX21 (Supplementary Fig. 6C). These data imply an important role of the PABPC1-PGAM1 axis in circDDX21-accelerated cell proliferation.

By using a xenograft mouse model, knockdown of circDDX21 was shown to significantly inhibit in vivo xenograft tumor growth of HepG2 cells (Fig. 6I–L). In contrast, overexpression of circDDX21 in HepG2 cells substantially enhanced in vivo xenograft tumor growth (Supplementary Fig. 6D–G). CircDDX21 knockdown-reduced xenograft tumor growth was markedly recovered by PGAM1 overexpression (Fig. 6I-L). The enhancing effect of circDDX21 on xenograft tumor growth was completely diminished by PGAM1 knockdown (Supplementary Fig. 6D–G). These data suggest that circDDX21 facilitates in vivo hepatocellular carcinoma cell growth via PGAM1. To further validate the clinical implication of circDDX21 in hepatocellular carcinogenesis, we examined the expression levels of circDDX21 in hepatocellular carcinoma tissues and matched adjacent normal tissues. Compared to the normal tissue samples, over 70% (18 of 25) of hepatocellular carcinoma (HCC) tissue samples exhibited elevated expression of circDDX21 (Fig. 6M). Furthermore, circDDX21 expression was positively correlated with PGAM1 expression in all the examined hepatocellular carcinoma tissue samples (n = 51) (Fig. 6N), indicating the biological importance of the circDDX21-PGAM1 axis in hepatocellular carcinogenesis. Taken together, these data strongly support that circDDX21 functions as an oncogenic circRNA in hepatocellular carcinoma.

## Discussion

Cancer cells actively rewire their metabolism in response to energy stress conditions in the tumor microenvironment [51, 52]. Increased glycolysis has been recognized as one of the major hallmarks of cancer cells [2]. However, it remains unknown whether energy stress-responsive circRNA plays a regulatory role in glycolysis. In this study, we report that circDDX21, as a glucose deprivation-induced circRNA, promotes glycolysis by increasing the expression of the glycolytic enzyme PGAM1. Therefore, circDDX21 may represent an important molecule that links glycolysis to the glucose-deprived microenvironment of tumors.

As a master transcription factor, c-Myc has been implicated in the regulation of cell metabolism by modulating target gene expression under both non-stressed and stressed conditions [53]. For instance, c-Myc is activated under energy stress conditions, which in turn upregulates the expression of key genes in the serine synthesis pathway and results in increased serine synthesis [54]. Here, we present evidence showing that c-Myc is responsible for the increased expression of circDDX21 in response to glucose deprivation. This finding, together with the critical role of circDDX21 in promoting glycolysis, suggests that circRNAs may be an important class of molecules that connect c-Myc to metabolic reprogramming in cancer cells.

The glycolytic enzyme PGAM1 plays a critical role in cancer metabolism by coordinating glycolysis and biosynthesis to promote rapid tumor growth [16]. Given the importance of PGAM1 in regulating cancer metabolism, the expression and activity of PGAM1 are not surprisingly controlled at different levels. For instance, PGAM1 is transcriptionally regulated by the transcription factors p53 and HIF-1α [20, 24]. PGAM1 is also subjected to post-translational modifications such as acetylation and phosphorylation, which affect its enzymatic activity [22, 23]. Moreover, the lncRNAs NEAT1 and glycoLINC can act as scaffold molecules for PGAM1 to interact with other glycolytic enzymes, thereby ensuring the efficiency of glycolysis [55, 56]. Here, we show that, correlating with its predominant cytoplasmic localization, circDDX21 is able to promote PGAM1 mRNA stability, indicating that circDDX21 is an important factor that finely controls PGAM1 expression. These findings also highlight the complexity of PGAM1 regulation.

CircRNAs have been shown to regulate gene expression via multiple mechanisms. One of the well-accepted mechanisms is circRNA-mediated gene regulation through interaction with their target proteins [57]. Here, we show that circDDX21 cooperates with the RNA binding protein PABPC1 to stabilize PGAM1 mRNA. PABPC1 belongs to the PABP family of proteins that bind poly-A or AU-rich sequences in mRNAs [58]. PABPC1 is able to regulate different aspects of RNA metabolism, including mRNA stability and translation [59–61]. CircDDX21 is identified as a new PABPC1 binding partner. The putative PABPC1-binding site within circDDX21 appears to be necessary for the interaction with PABPC1. We also show that by binding to PABPC1, circDDX21 enhances the association between PABPC1 and PGAM1 mRNA, thereby stabilizing PGAM1 mRNA. It has been previously reported that PABPC1 undergoes MKRN3-mediated ubiquitination, which inhibits the binding of PABPC1 to its target mRNA [49, 50]. Intriguingly, circDDX21 appears to compete with MKRN3 for binding to PABPC1 and suppresses MKRN3-mediated PABPC1 ubiquitination, thereafter increasing the interaction of PABPC1 with PGAM1 mRNA. These findings suggest an important role of the MKRN3-PABPC1 axis in mediating the promoting effect of circDDX21 on PGAM1 mRNA stability. It has been recently reported that circDDX21 functions as a sponge for miR-1264 to regulate QKI expression in triple-negative breast cancer [62]. Therefore, it would be interesting to investigate whether circDDX21 could also act as a miRNA sponge to regulate PGAM1 mRNA stability in the future.

Overexpression of PGAM1 has been found in various human cancers, and increased expression of PGAM1 is associated with poor prognosis in cancer patients [19, 63]. In this study, we show that consistent with the enhancing effect of circDDX21 on PGAM1 expression, circDDX21 is able to promote both in vitro hepatocellular carcinoma cell proliferation and in vivo xenograft tumor growth via PGAM1. In addition, circDDX21 is highly expressed in clinical hepatocellular carcinoma tissues compared to normal tissues. The expression of circDDX21 and PGAM1 are also positively correlated in hepatocellular carcinoma. These data support that circDDX21 functions as an oncogenic circRNA in hepatocellular carcinoma. Unlike this oncogenic function in hepatocellular carcinoma, circDDX21 seems to play a tumor-suppressive role in triple-negative breast cancer [62], indicating that circDDX21 may function in vivo as an oncogene or tumor suppressor in different cancer types. Taken together, our study demonstrates an important role of circDDX21 in promoting glycolysis and hepatocellular carcinogenesis, and suggests that circDDX21 may represent a potential therapeutic target for hepatocellular carcinoma.

### Supplementary information

Supplementary Figures and Tables

Uncropped original western blots

## Data Availability

The authors confirm that the data supporting the findings of this study are available within the article and its supplementary materials.
