# Supplementary material for: Energy stress-induced circDDX21 promotes glycolysis and facilitates hepatocellular carcinogenesis
Source: Cell Death Dis. 2024 May 21;15(5):354. doi: 10.1038/s41419-024-06743-1 (PMC11109331; doi:10.1038/s41419-024-06743-1)
Supplement: Supplementary file 1 — Supplementary Figures and Tables [file 41419_2024_6743_MOESM1_ESM.pdf]

Supplementary Figure Legends

Figure S1. Related to Figure 1.

(A) The knockdown efficiency of the indicated shRNAs was assessed by real-time RT-PCR.

(B) Shown are the copy numbers of circDDX21 per HepG2 cell cultured in glucose-deprived (GD) medium for the indicated periods of time.

(C) Shown are the copy numbers of circDDX21 per PLC cell cultured in glucose-deprived (GD) medium for the indicated periods of time.

(D) Real-time RT-PCR analysis of DDX21 and circDDX21 levels in PLC cells treated with or without actinomycin D (2 μg/ml) for 12 h. Data shown are mean ± SD (n=3), $^{**}p < 0.01$, ns., no significance.

(E) Real-time RT-PCR analysis of DDX21 and circDDX21 levels in total RNA from PLC cells after treatment with or without RNase R (3 U/μg) for 10 min. Data shown are mean ± SD (n=3), $^{***}p < 0.001$, ns., no significance.

(F) Fluorescence in situ hybridization (FISH) detection of circDDX21 in HepG2 cells.

(G) The cytoplasmic and nuclear fractions from HepG2 cells were analyzed by real-time RT-PCR.

(H) Schematic illustration of how the circRNA translation GFP reporter system works. The possibility of circDDX21 translation was evaluated by immunofluorescence and western blotting analyses 24 h after transfection of the reporter plasmids into HEK293T cells.

Figure S2. Related to Figure 2.

(A) Real-time RT-PCR analysis of pre-DDX21 and circDDX21 levels in HepG2 cells cultured in glucose-deprived (GD) medium for the indicated periods of time. Data shown are mean ± SD (n=3), $^{**}p < 0.01$.

(B) Real-time RT-PCR analysis of pre-DDX21 and circDDX21 levels in PLC cells cultured in glucose-deprived (GD) medium for the indicated periods of time. Data shown are mean ± SD (n=3), $^{***}p < 0.001$.

(C) Schematic illustration of three transcription factors predicted by the JASPAR database that potentially bind to the 1,000 bp region around the transcription start site of DDX21.

(D and E) HepG2 cells were infected with lentiviruses expressing control shRNA, c-Myc shRNA, NRF1 shRNA, or YY1 shRNA. (D) Forty-eight hours after infection, cells were cultured in normal or glucose deprived medium for 24 h as indicated, followed by real-time RT-PCR analysis to examine circDDX21 levels. (E) The knockdown efficiency of the indicated genes was also examined by real-time RT-PCR analysis. Data shown are mean ± SD (n=3), $*p < 0.05$, $***p < 0.001$, ns., no significance.

(F) Real-time RT-PCR analysis of pre-DDX21 and circDDX21 levels in PLC cells expressing control shRNA or c-Myc shRNA. Data shown are mean ± SD (n=3), $**p < 0.01$. The knockdown effiency of c-Myc was also evaluated by western blotting.

(G) Real-time RT-PCR analysis of pre-DDX21 and circDDX21 levels in PLC cells with or without ectopic expression of c-Myc. Data shown are mean ± SD (n=3), $***p < 0.001$. The successful overexpression of c-Myc was also evaluated by western blotting.

Figure S3. Related to Figure 3.

(A) Western blot analysis of lysates from HepG2 cells transduced with lentiviruses expressing empty vector (EV) or circDDX21.

(B) Western blot analysis of lysates from PLC cells transduced with lentiviruses expressing control shRNA, circDDX21 shRNA#1, or circDDX21 shRNA#2.

(C) Real-time RT-PCR analysis of circDDX21 and DDX21 levels in PLC cells transduced with lentiviruses expressing control shRNA, circDDX21 shRNA#1, or circDDX21 shRNA#2. Data shown are mean ± SD (n=3), $**p < 0.01$, $***p < 0.001$, ns., no significance.

(D) Western blot analysis of lysates from PLC cells transduced with lentiviruses expressing empty vector (EV) or circDDX21.

(E) Real-time RT-PCR analysis of circDDX21 and DDX21 levels in PLC cells transduced with lentiviruses expressing empty vector (EV) or circDDX21. Data shown are mean ± SD (n=3), $**p < 0.01$, ns., no significance.

(F) Real-time RT-PCR analysis of DDX21 and PGAM1 RNA levels in HepG2 cells with knockdown or overexpression of DDX21. Data shown are mean ± SD (n=3), ns, no significance.

(G) Western blot analysis of PGAM1 protein levels in HepG2 cells with knockdown or overexpression of DDX21.

Figure S4. Related to Figure 4.

(A) Real-time RT-PCR analysis of PGAM1 mRNA levels in PLC cells transduced with lentiviruses expressing control shRNA, circDDX21 shRNA#1, or circDDX21 shRNA#2. Data shown are mean ± SD (n=3), $^{**}p < 0.01$.

(B) Real-time RT-PCR analysis of PGAM1 mRNA levels in PLC cells transduced with lentiviruses expressing empty vector (EV) or circDDX21. Data shown are mean ± SD (n=3), $^{**}p < 0.01$.

(C) PLC cells transduced with lentiviruses control shRNA, circDDX21 shRNA#1, or circDDX21 shRNA#2 were incubated with actinomycin D (2 μg/mL) for the indicated periods of time, followed by real-time RT-PCR analysis to examine PGAM1 mRNA stability. Data shown are mean ± SD (n=3), $^{*}p < 0.05$, $^{**}p < 0.01$.

(D and E) (D) Potential circDDX21-binding proteins pulled down by biotin-labeled antisense DNA oligos against circDDX21 were separated by SDS-PAGE, followed by mass spectrometry analysis. (E) Shown are the top 10 identified potential circDDX21-binding proteins.

(F) Real-time RT-PCR analysis of PGAM1 mRNA levels in HepG2 cells transduced with lentiviruses expressing the indicated shRNAs.

(G) Real-time RT-PCR and western blot analyses of PGAM1 expression levels in HepG2 cells transduced with lentiviruses expressing the indicated shRNAs. Data shown are mean ± SD (n=3), $^{**}p < 0.01$, $^{***}p < 0.001$.

(H) HepG2 cells transduced with lentiviruses control shRNA or PABPC1 shRNA were incubated with actinomycin D (2 μg/mL) for the indicated periods of time, followed by real-time RT-PCR analysis to examine PGAM1 mRNA stability. Data shown are mean ± SD (n=3), $^{**}p < 0.01$.

(I) Schematic illustrating cyclization of linear RNA generated in vitro. RT-PCR analysis of linear and cyclized circEPB41(2) RNAs with divergent primers.

(J) Shown is the circDDX21 mutant (ΔPABPC1 BS) with mutations in the potential PABPC1-binding site.

(K) Purified recombinant Flag-PABPC1 bound with M2 beads was incubated with in vitro synthesized circDDX21 or the circDDX21 mutant (ΔPABPC1 BS). The bead-bound RNAs were then analyzed by RT-PCR.

(L) Real-time RT-PCR analysis of PGAM1 mRNA levels in PLC cells transduced with lentiviruses expressing control, circDDX21 shRNA, PABPC1, or both circDDX21 shRNA and PABPC1. Data shown are mean ± SD (n=3), $^{**}p < 0.01$, $^{***}p < 0.001$.

(M) Western blot analysis of PGAM1 protein levels in PLC cells transduced with lentiviruses expressing control, circDDX21 shRNA, Flag-PABPC1, or both circDDX21 shRNA and Flag-PABPC1.

(N) Real-time RT-PCR analysis of PGAM1 mRNA levels in PLC cells transduced with lentiviruses expressing control, circDDX21, PABPC1 shRNA, or both circDDX21 and PABPC1 shRNA. Data shown are mean ± SD (n=3), $^{**}p < 0.01$, $^{***}p < 0.001$, ns., no significance.

(O) Western blot analysis of PGAM1 protein levels in PLC cells transduced with lentiviruses expressing control, circDDX21, PABPC1 shRNA, or both circDDX21 and PABPC1 shRNA.

(P) Schematic illustration of the psiCHECK2-based PGAM1 3'-UTR reporter construct used for luciferase assay.

Figure S5. Related to Figure 5.

(A) Lysates from HepG2 cells transduced with lentiviruses expressing empty vector (EV), circDDX21, or mutant circDDX21 (ΔPABPC1 BS) were subjected to an in vivo ubiquitination assay using anti-PABPC1 antibody.

(B) Schematic illustration of the functional domains of PABPC1.

(C) Purified recombinant Flag-PABPC1 or its truncated mutants bound with M2 beads was incubated with in vitro synthesized circDDX21. The bead-bound RNAs were then analyzed by RT-PCR.

Figure S6. Related to Figure 6.

(A) Growth curves of HepG2 cells expressing control, circDDX21, or circDDX21 mutant (ΔPABPC1 BS). Data shown are mean ± SD ($n = 3$). **$p < 0.01$, ns, no significance.

(B) Colonies of HepG2 cells expressing control, circDDX21, or circDDX21 mutant (ΔPABPC1 BS) were stained with crystal violet after 14 days of incubation. Data shown are mean ± SD ($n = 3$). **$p < 0.01$, ns, no significance.

(C) ECAR was measured by Seahorse XF assay in HepG2 cells expressing empty vector (EV), circDDX21, or mutant circDDX21 (ΔPABPC1 BS). The levels of glycolysis, glycolytic capacity, and glycolytic reserve were also calculated. Data shown are mean ± SD ($n=3$), **$p < 0.01$, ***$p < 0.001$, ns, no significance.

(D-G) A total of 3×10^6 HepG2 cells expressing control, circDDX21, PGAM1 shRNA, or both circDDX21 and PGAM1 shRNA were individually injected into nude mice ($n = 6$ for each group). (D) Xenograft tumors were taken 24 days after injection. (E) Excised tumors were weighed. **$p < 0.01$, ns, no significance. (F) Tumor sizes were measured at the indicated time points. **$p < 0.01$, ***$p < 0.001$, ns, no significance. (G) RNA and protein extracts from the excised xenografts were analyzed by RT-PCR and western blotting, respectively.

Figure S1

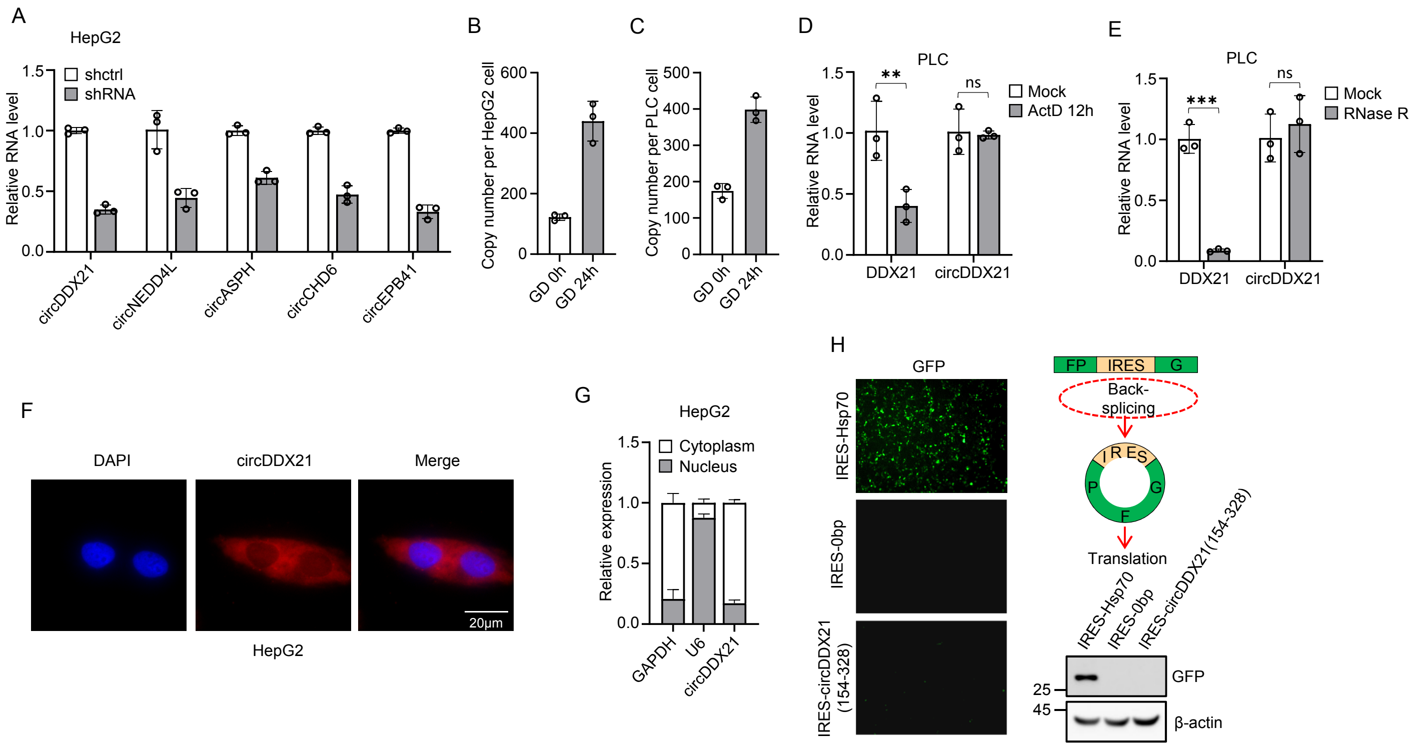
A
HepG2
shctrl
shRNA
Relative RNA level
circDDX21
circNEDD4L
circASPH
circCHD6
circEPB41
B
Copy number per HepG2 cell
GD 0h
GD 24h
C
Copy number per PLC cell
GD 0h
GD 24h
D
PLC
**
ns
Mock
ActD 12h
Relative RNA level
DDX21
circDDX21
E
PLC
***
ns
Mock
RNase R
Relative RNA level
DDX21
circDDX21
F
DAPI
circDDX21
Merge
20μm
HepG2
G
HepG2
Cytoplasm
Nucleus
Relative expression
GAPDH
U6
circDDX21
H
GFP
IRES-Hsp70
IRES-0bp
IRES-circDDX21 (154-328)
FP
IRES
G
Back-splicing
Translation
IRES-Hsp70
IRES-0bp
IRES-circDDX21(154-328)
25
45
GFP
β-actin

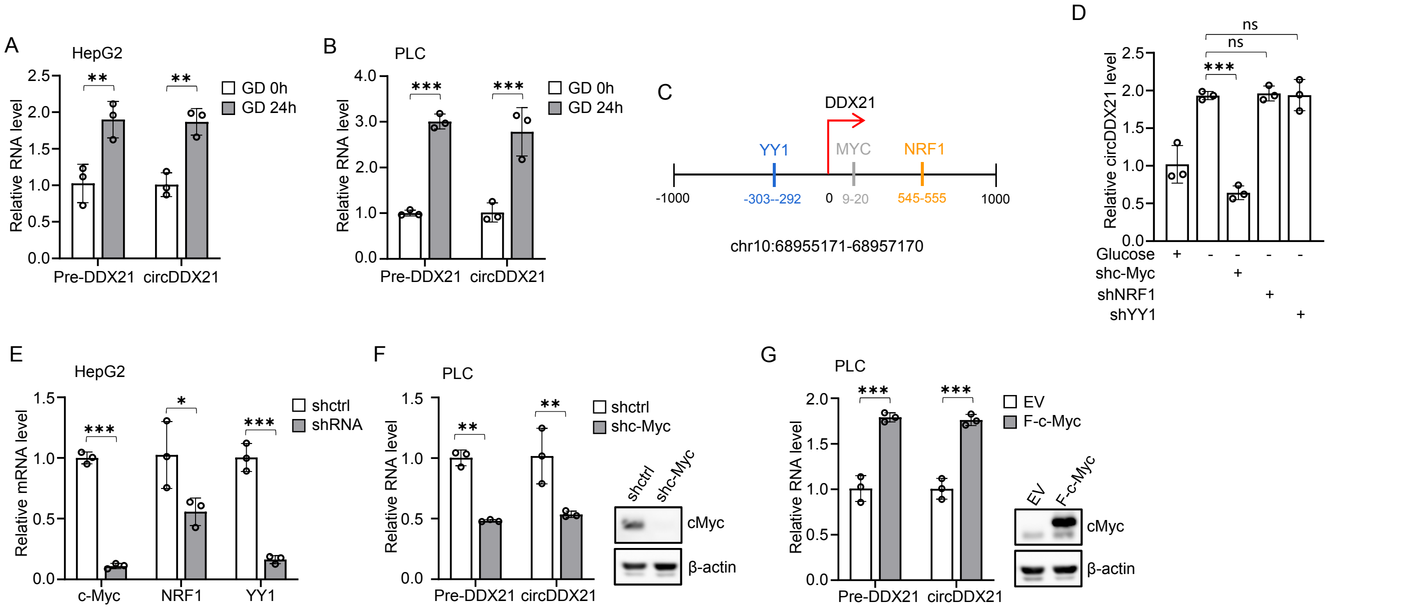
A
HepG2
Relative RNA level
GD 0h
GD 24h
Pre-DDX21
circDDX21
B
PLC
Relative RNA level
GD 0h
GD 24h
Pre-DDX21
circDDX21
C
DDX21
YY1
MYC
NRF1
-1000
-303--292
0
9-20
545-555
1000
chr10:68955171-68957170
D
Relative circDDX21 level
ns
ns
Glucose
shc-Myc
shNRF1
shYY1
E
HepG2
Relative mRNA level
shctrl
shRNA
c-Myc
NRF1
YY1
F
PLC
Relative RNA level
shctrl
shc-Myc
cMyc
β-actin
Pre-DDX21
circDDX21
G
PLC
Relative RNA level
EV
F-c-Myc
cMyc
β-actin
Pre-DDX21
circDDX21

Figure S3

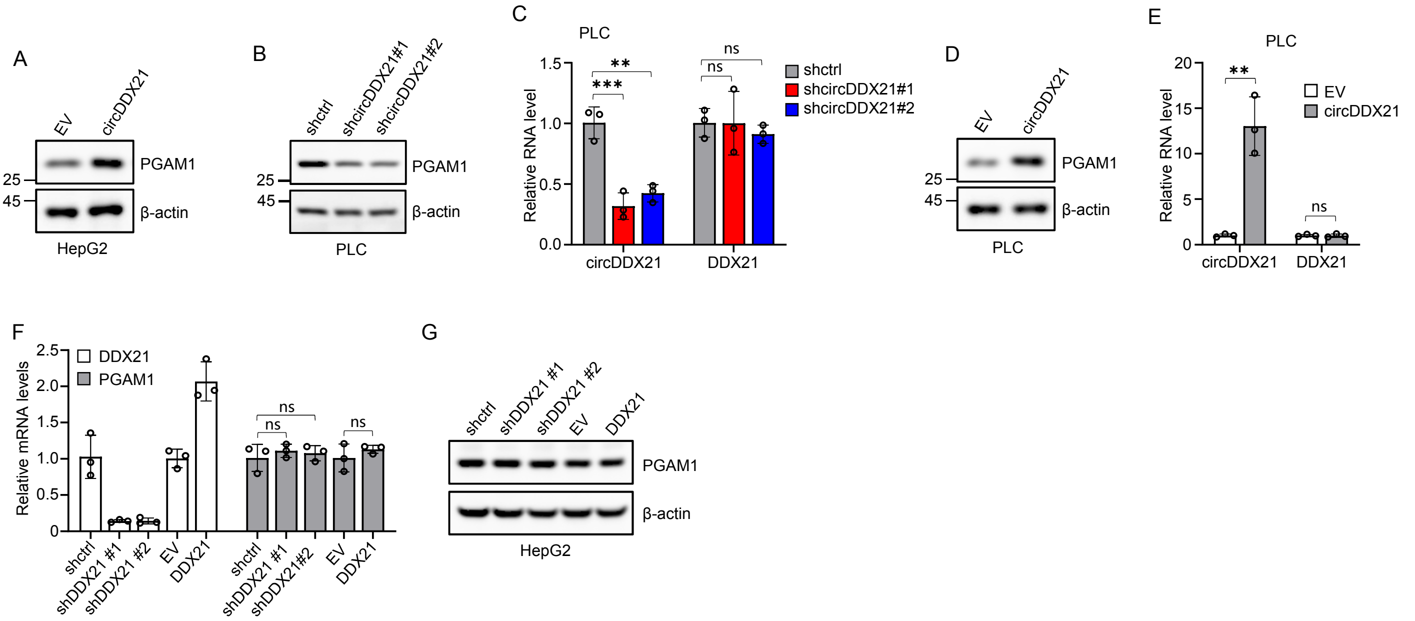
A
EV
circDDX21
PGAM1
β-actin
25
45
HepG2
B
shctrl
shcircDDX21#1
shcircDDX21#2
PGAM1
β-actin
PLC
C
PLC
Relative RNA level
shctrl
shcircDDX21#1
shcircDDX21#2
circDDX21
DDX21
ns
**
***
D
EV
circDDX21
PGAM1
β-actin
PLC
E
PLC
Relative RNA level
EV
circDDX21
**
ns
F
Relative mRNA levels
DDX21
PGAM1
shctrl
shDDX21 #1
shDDX21 #2
EV
DDX21
ns
G
shctrl
shDDX21 #1
shDDX21 #2
EV
DDX21
PGAM1
β-actin
HepG2

Figure S4

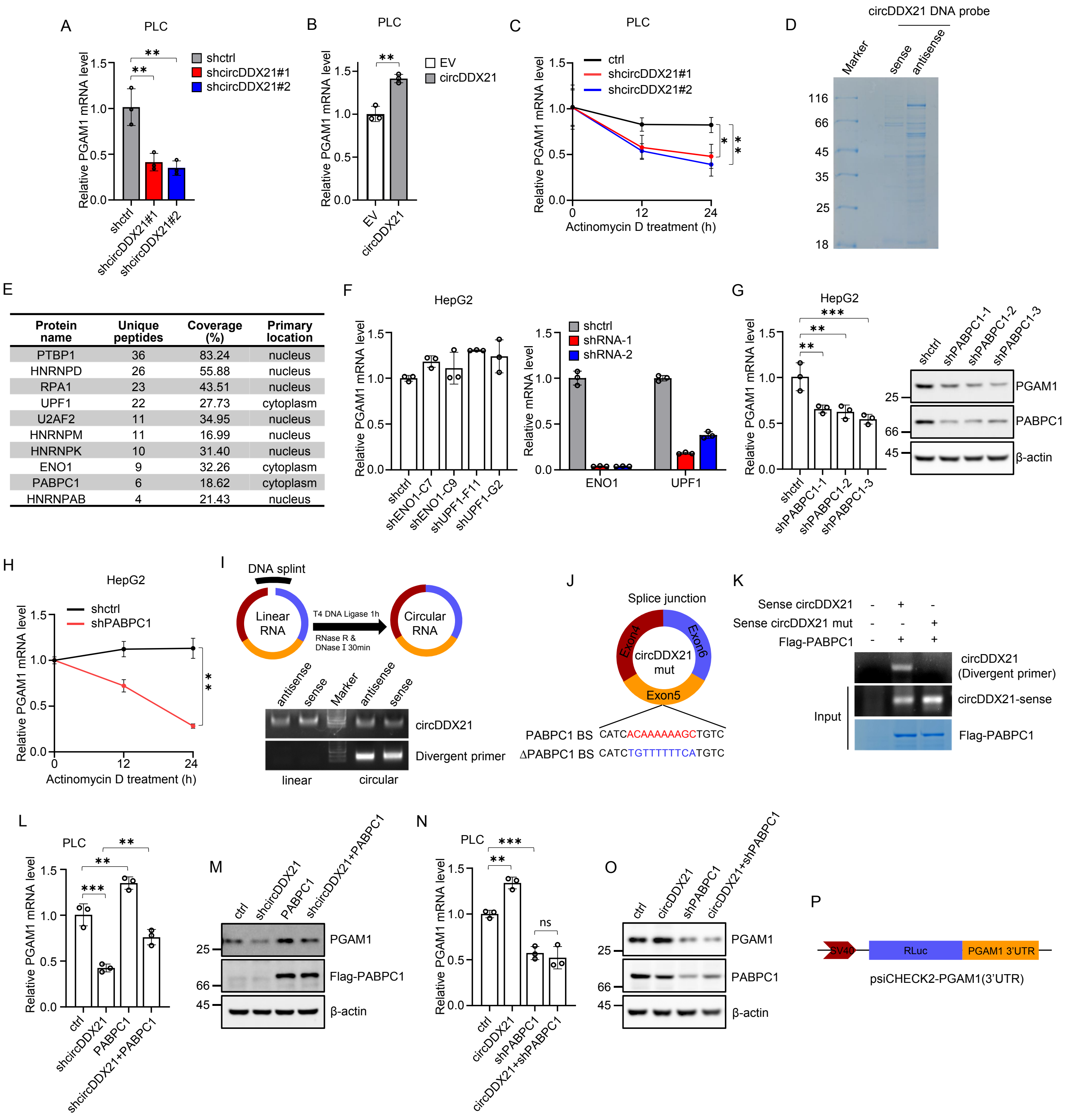

Protein name	Unique peptides	Coverage (%)	Primary location
PTBP1	36	83.24	nucleus
HNRNPD	26	55.88	nucleus
RPA1	23	43.51	nucleus
UPF1	22	27.73	cytoplasm
U2AF2	11	34.95	nucleus
HNRNPM	11	16.99	nucleus
HNRNPK	10	31.40	nucleus
ENO1	9	32.26	cytoplasm
PABPC1	6	18.62	cytoplasm
HNRNPAB	4	21.43	nucleus

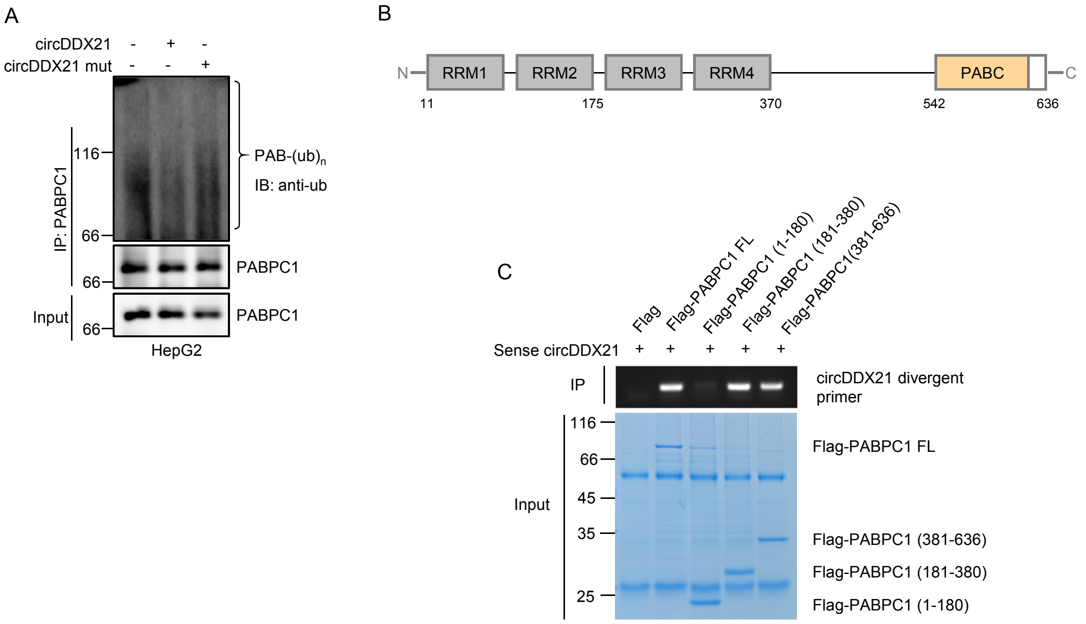
A
circDDX21 - + -
circDDX21 mut - - +
IP: PABPC1
116
66
PAB-(ub)n
IB: anti-ub
66
PABPC1
Input
66
PABPC1
HepG2
B
N
RRM1
RRM2
RRM3
RRM4
PABC
C
11
175
370
542
636
C
Flag
Flag-PABPC1 FL
Flag-PABPC1 (1-180)
Flag-PABPC1 (181-380)
Flag-PABPC1(381-636)
Sense circDDX21 + + + + +
IP
circDDX21 divergent primer
116
66
45
35
25
Input
Flag-PABPC1 FL
Flag-PABPC1 (381-636)
Flag-PABPC1 (181-380)
Flag-PABPC1 (1-180)

Figure S6

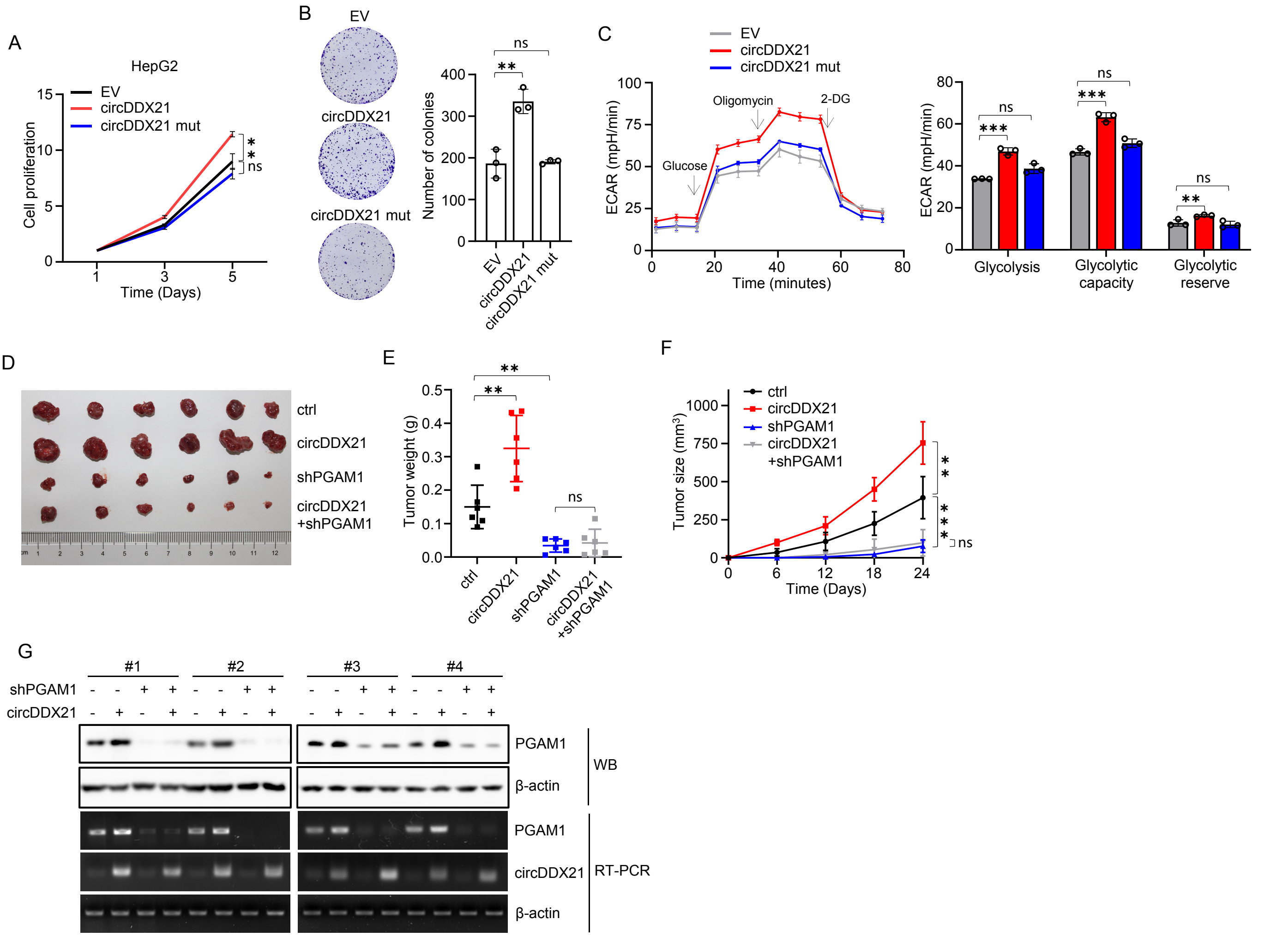

A
HepG2
EV
circDDX21
circDDX21 mut
Cell proliferation
Time (Days)
B
EV
circDDX21
circDDX21 mut
Number of colonies
C
EV
circDDX21
circDDX21 mut
ECAR (mpH/min)
Glucose
Oligomycin
2-DG
Time (minutes)
Glycolysis
Glycolytic capacity
Glycolytic reserve
D
ctrl
circDDX21
shPGAM1
circDDX21
+shPGAM1
E
Tumor weight (g)
F
ctrl
circDDX21
shPGAM1
circDDX21
+shPGAM1
Tumor size (mm³)
Time (Days)
G
#1
#2
#3
#4
shPGAM1
circDDX21
PGAM1
β-actin
WB
PGAM1
circDDX21
β-actin
RT-PCR

Supplementary Table 1. 28 up-regulated circRNAs upon glucose deprivation

Candidate	CircBase ID	RNA length	chromosome	log2Fold Change	p value
circATAD2	hsa_circ_0085465	333	chr8	6.2614	0.0008
circRNF145	hsa_circ_0074828	332	chr5	5.8635	0.0016
circSND1	hsa_circ_0003655	312	chr7	5.5717	0.0031
circDDX21	hsa_circ_0008523	483	chr10	5.4504	0.0041
circATP9A	hsa_circ_0008253	259	chr20	5.2317	0.0074
circSMARCC1	hsa_circ_0005367	381	chr3	4.9936	0.0105
circILKAP	hsa_circ_0058876	781	chr2	4.941	0.0115
circRSRC1	hsa_circ_0067808	496	chr3	4.9218	0.0120
circNEDD4L	hsa_circ_0007510	642	chr18	4.5782	0.0218
circASPH	hsa_circ_0006755	150	chr8	4.4737	0.0258
circUSO1	hsa_circ_0007096	244	chr4	4.4737	0.0258
circCHD6	hsa_circ_0060423	521	chr20	4.4737	0.0258
circEPB41	hsa_circ_0011167	475	chr1	4.4497	0.0283
circCDC73	hsa_circ_0015749	445	chr1	4.4224	0.0279
circNUP107	hsa_circ_0027477	765	chr12	4.3955	0.0292
circRPE	hsa_circ_0007225	1812	chr2	4.359	0.0309
circCDKL4	novel_circ_0017711	224	chr2	4.3525	0.0312
circAGAP1	hsa_circ_0003272	321	chr2	4.2982	0.0338
circSCFD1	novel_circ_0005388	302	chr14	4.2982	0.0338
circCUL3	novel_circ_0017068	279	chr2	4.2537	0.0364
circSMTN	hsa_circ_0002149	394	chr22	4.2321	0.0374
circARID4B	novel_circ_0012626	258	chr1	4.2321	0.0374
circUBE2Q2	hsa_circ_0036377	497	chr15	4.1592	0.0415
circR3HDM1	novel_circ_0016077	149	chr2	4.1508	0.0422
circRUFY2	novel_circ_0000982	168	chr10	4.0901	0.0458
circFOXO1	novel_circ_0004823	148	chr13	4.0901	0.0458
circSNX14	hsa_circ_0008991	217	chr6	4.055	0.0483
circSNX14	novel_circ_0024385	112	chr6	4.055	0.0483

Supplementary Table 2. Oligonucleotides used in this study

**Oligonucleotide sequence of shRNAs**	
sh-circDDX21#1	5'GAAAGGCCGAGGAGTGACCTT3'
sh-circDDX21#2	5'AAGAAAGGCCGAGGAGTGACC3'
sh-circNEDD4L	5'ACTGGTCCGCCATCAGTGGCC3'
sh-circCHD6	5'GTCCAGGAAGGCCAGTTGTCA3'
sh-circASPH	5'GAGGAAGTTCTAGAGACAAAG3'
sh-circEPB41	5'GCAGAAACACAGCAACATCAT3'
sh-c-Myc	5'CTGAGACAGATCAGCAACAA3'
sh-NRF1	5'CCGTTGCCCAAGTGAATTATT3'
sh-YY1	5'GCCTCTCCTTTGTATATTATT3'
sh-PGAM1	5'CCGGTCTCAATAAAGCAGAAA3'
sh-PABPC1-1	5'CCAGACCTCATCCATTCCAAA3'
sh-PABPC1-2	5'CCGCACCGTTCCACAGTATAA3'
sh-PABPC1-3	5'AGGCGATGCTCTACGAGAAGT3'
sh-ENO1-C7	5'CGTACCGCTTCCTTAGAACTT3'
sh-ENO1-C9	5'GAATGTCATCAAGGAGAAATA3'
sh-UPF1-F11	5'GCATCTTATTCTGGGTAATAA3'
sh-UPF1-G2	5'CCAACCCGATAAACCGATGTT3'
sh-DDX21#1	5'GCGGAGTTTCAGTAAAGCATT3'
sh-DDX21#2	5'CCCATATCTGAAGAAACTATT3'
**Oligonucleotide sequence of sgRNA**	
sg-MKRN3	5'TATTAGGTGTATCCGCAGG3'
**Primers used in qRT-PCR analysis & Semi-quantitative RT-PCR**	
β-Actin	Fw: 5'GACCTGACTGACTACCTCATGAAGAT3'
	Rev: 5'GTCACACTTCATGATGGAGTTGAAGG3'
circATAD2	Fw: 5'AAGCCTGTTGACCCTGATGA3'
	Rev: 5'GGAGGCTTAGCAGCTTGTTTT3'
circRNF145	Fw: 5'TGCTCTGCTCCTCTATGCTG3'
	Rev: 5'AGGGCCACATTTAACACTGC3'
circSND1	Fw: 5'CAAGACGATTCACCTGTCCA3'
	Rev: 5'AAACTGCAATCGACCAGTCC3'
circDDX21	Fw: 5'GGGATTTGCTGATCAAGTGGAAG3'
	Rev: 5'AGTTCCTGTCCGTGCCTGT3'
circATP9A	Fw: 5'TTCCCGAAATGAGACTTGGT3'
	Rev: 5'CCGAGGATACCTCTGGTCTCT3'
circSMARCC1	Fw: 5'TGACCAGACCCAACATCTACC3'
	Rev: 5'CAGCCCAGCCAGTGTTTTAT3'
circILKAP	Fw: 5'GCCTTAAGCCTCAGCAAAGA3'
	Rev: 5'ATCAAAGAGCAGGGGTCCTT3'
circRSRC1	Fw: 5'GAACGACGTAAGGGCAGAGA3'
	Rev: 5'AGGACCGTCTACGGTGTTTC3'

circNEDD4L	Fw: 5'CTCAGTGGGAAGACCCAAGA3'
	Rev: 5'TAGTATGTGCGCCCCTTAGC3'
circASPH	Fw: 5'GGCGTCTGGACATCTGTAGC3'
	Rev: 5'CCACGTGAAGAATGAAGTTCC3'
circUSO1	Fw: 5'AAAGGCAGCCATTTGTTTTG3'
	Rev: 5'TTTACTTGGCAGCCTCGAAT3'
circCHD6	Fw: 5'AGGAGGCCAAAGAGAAGAGG3'
	Rev: 5'CAGTCAAATGGGGATGGAGA3'
circEPB41	Fw: 5'CGGAACTCAAAACAGACCCA3'
	Rev: 5'CGGCCTCAGTCACTAAACTC3'
circCDC73	Fw: 5'TTGTAGCCGTTTTTGTGCAG3'
	Rev: 5'TCTGATTTGGGGGAGGTCTT3'
circNUP107	Fw: 5'TGCTTCGCTTTATGACTCACC3'
	Rev: 5'CAGATCATCAAGGGGCATTT3'
circDDX21 Convergent	Fw: 5'CATTCTCCTTTGCCATCCC'
	Rev: 5'GTCTTTGCTTACTTGATTTGCC3'
β-Actin Divergent	Fw: 5'AAAGGCGAGGCTCTGTGCT3'
	Rev: 5'GGGCTTACCTGTACACTGACTGA3'
CDR1as Divergent	Fw: 5'ACGTCTCCAGTGTGCTGA3'
	Rev: 5'CTTGACACAGGTGCCATC3'
CDR1as Convergent	Fw: 5'TTTCCGATGGCACCTGTGTCAAG3'
	Rev: 5'CTGGAAGACCTTGAGATTATTGGAAGAC3'
DDX21	Fw: 5'CGCATGAGGAATGGGATTGATA3'
	Rev: 5'CCACTTCATCCAGGACAACAT3'
GAPDH	Fw: 5'CCATGGGGAAGGTGAAGGTC3'
	Rev: 5'GAAGGGGTCATTGATGGCAAC3'
U6	Fw: 5'GCTTCGGCAGCACATATACTAAAAT3'
	Rev: 5'CGCTTCACGAATTTGCGTGTCAT3'
Pre-DDX21	Fw: 5'CACCCATTGTTGACTGCTCTG3'
	Rev: 5'TTGCCTATCATCAAGCAGGACC3'
c-Myc	Fw: 5'TAGTGGAAAACCAGCAGCCT3'
	Rev: 5'GGCAGCAGCTCGAATTTCTT3'
YY1	Fw: 5'TCAGATCCCAAACAACTGGCA3'
	Rev: 5'GGCCGAGTTATCCCTGAACA3'
NRF1	Fw: 5'GGTCGCAAGTGGATCCTGAC3'
	Rev: 5'GCGCTGTCTGATATCCTGGT3'
PGAM1	Fw: 5'GGGGCATTGTCAAGCATCTG3'
	Rev: 5'CTGCATGGGCTTGATAGGCT3'
ENO1	Fw: 5'CCTGCCCTGGTTAGCAAGAA3'
	Rev: 5'GGCGTTCGCACCAAACTTAG3'
UPF1	Fw: 5'CCAGCTCGCAGACTCTCAC3'

	Rev: 5'GCGTCTGGCTAGGAAGAGTAA3'
**Primers used in ChIP-qPCR assays**	
BS1	Fw: 5'TCTGGCCAGTGTTGAAGA3'
	Rev: 5'AGTGAATTGACCACTTGCGGCCCTTGGT3'
BS2	Fw: 5'TTTGAATTTACCACGCGGAAAGCAGCTT3'
	Rev: 5'GTAGTTCCCCACCCTGGG3'
BS3	Fw: 5'CTCTTCCTCTCCACGCGGTTGAGAAGAC3'
	Rev: 5'CTCCCCTTTTTTCATTGC3'
GAPDH promoter	Fw: 5'TACTAGCGGTTTTACGGGCG3'
	Rev: 5'TCGAACAGGAGGAGCAGAGAGCGA3'
**Oligonucleotides used for biotin pull-down assays**	
circDDX21 sense oligo DNA	5'TGGCATACAAGAAAGGCCGAGGAGTGACCT3'(-biotin)
circDDX21 antisense oligo DNA	5'AGGTCACTCCTCGGCCTTTCTTGTATGCCA3'(-biotin)
**Primers for In Vitro Cyclization**	
The T7 RNA polymerase sequence(T7) was 5'TAATACGACTCACTATAGGG3'	
Sense circDDX21	5'(T7)GCCGAGGAGTGACCTTCCT3'
	5'CTTTCTTGTATGCCACACT3'
Antisense circDDX21	5'GCCGAGGAGTGACCTTCCT3'
	5'(T7)CTTTCTTGTATGCCACACT3'
circDDX21 splint	ATAGGAAGGTCACTCCTCGGCCTTTCTTGTATGCCACACTTA
circDDX21-antisense splint	TAAGTGTGGCATACAAGAAAGGCCGAGGAGTGACCTTCCTAT
**Probe sequence used for RNA ISH**	
5'AAGGGATGGCAAAGGAGAATGTCTTCCCAGTTCCTGTCCGTGCCTGTGCAATTAAGTCCTTCCCGCTGTAAACATGATGGAATGTCTTTGCTTGTATAGGAAATAGGAAGGTCACTCCTCGGCCTTTCTTGTATGCCACACTTAAAATCTCTTCCACTTGATCAGCAAATCCCATATCCAACATCTGGTCCACTTCATCCAGGACAACA3'	
