# Supplementary figures and images for: Energy stress-induced circDDX21 promotes glycolysis and facilitates hepatocellular carcinogenesis
Source: Cell Death Dis. 2024 May 21;15(5):354. doi: 10.1038/s41419-024-06743-1 (PMC11109331; doi:10.1038/s41419-024-06743-1)

Fig.2

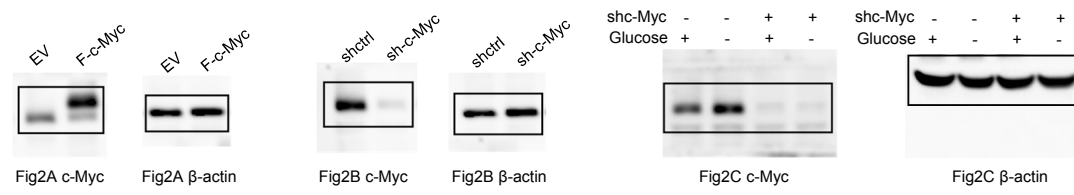

Fig.3

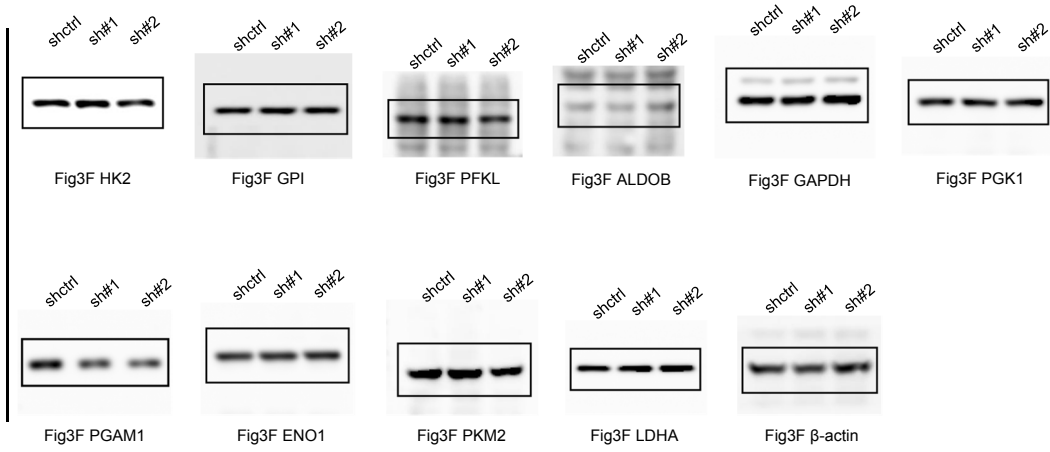

Fig.4

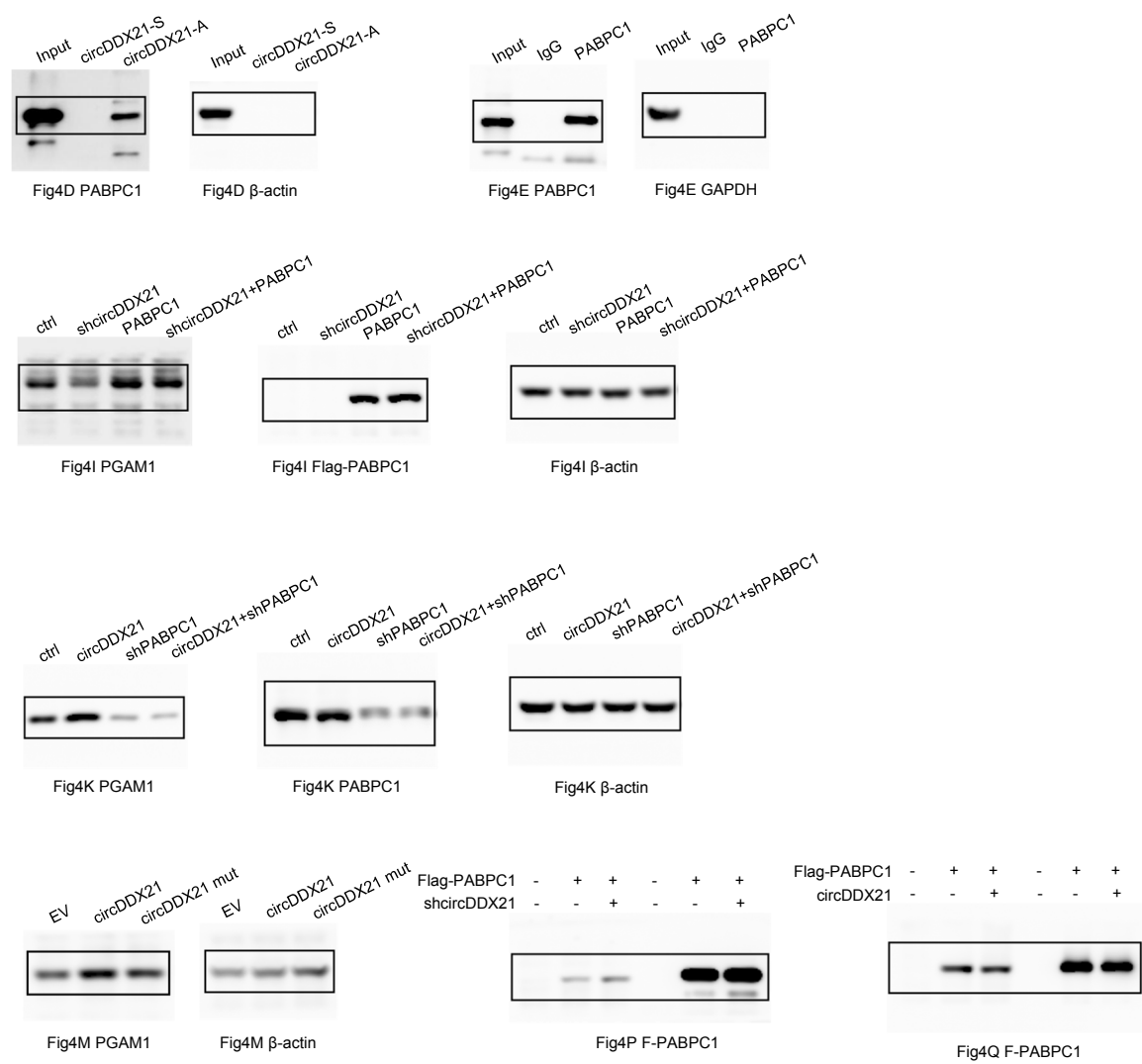

Fig.5

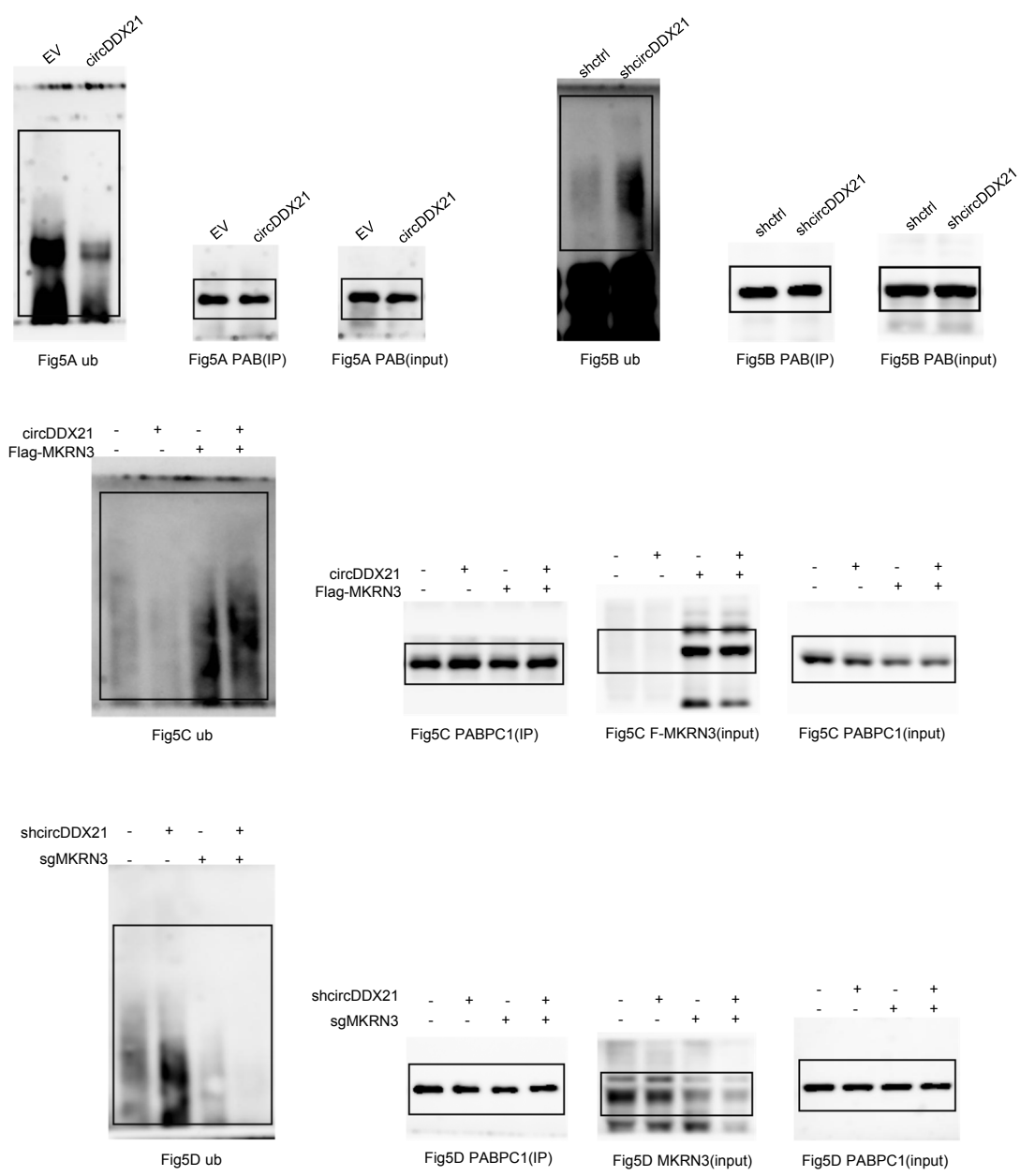

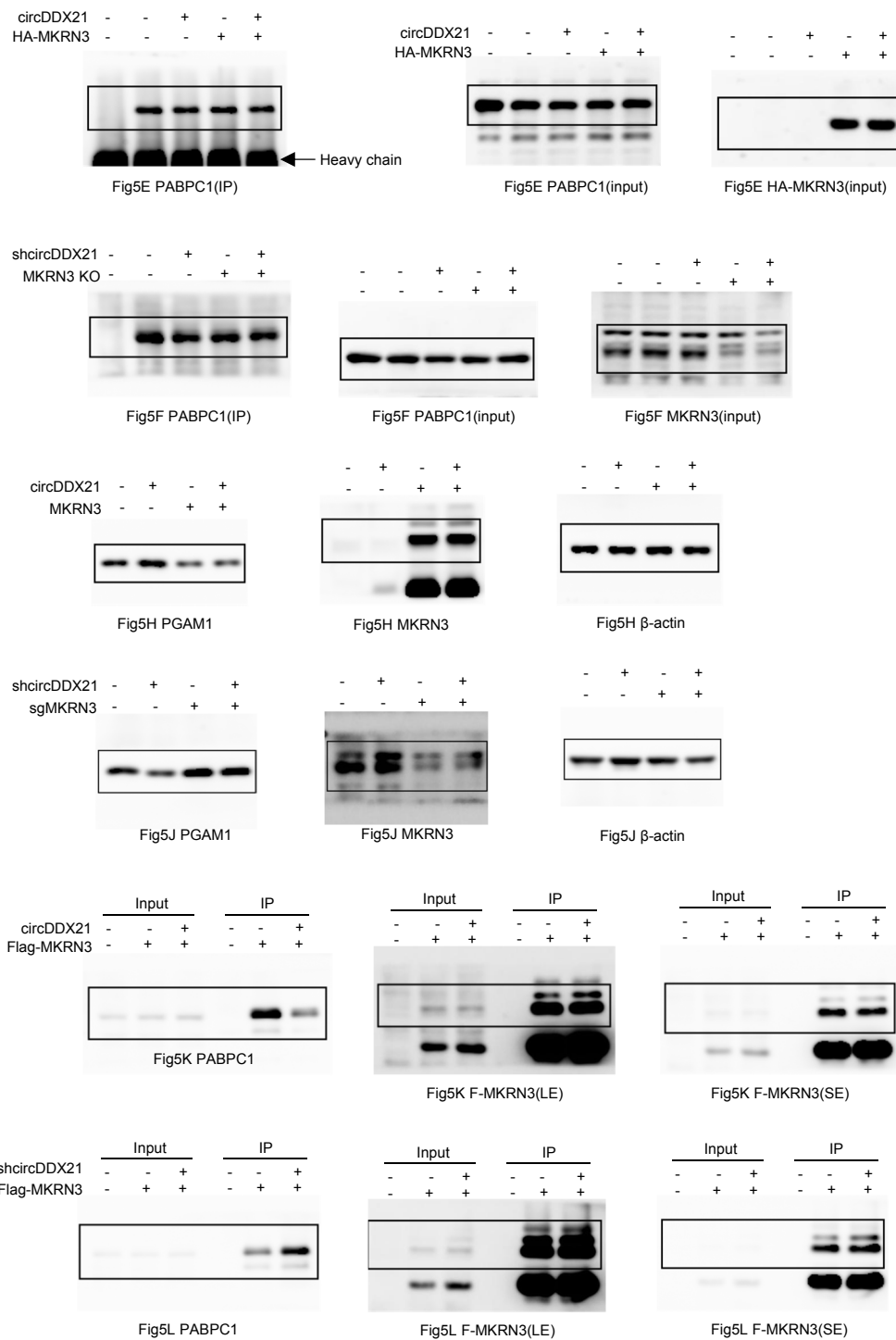

Fig.5

Fig.6

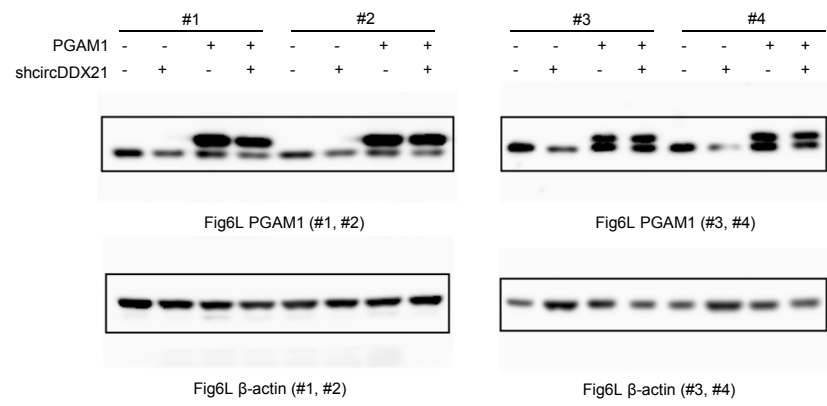

Fig. S1

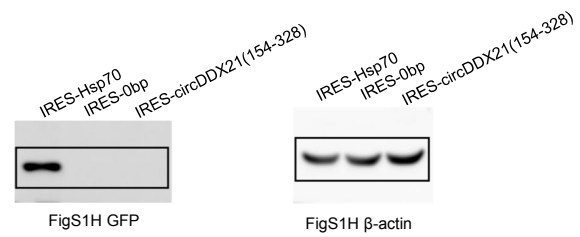

Fig. S2

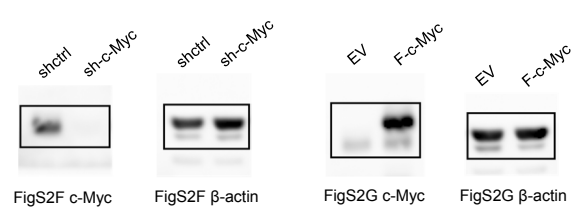

Fig. S3

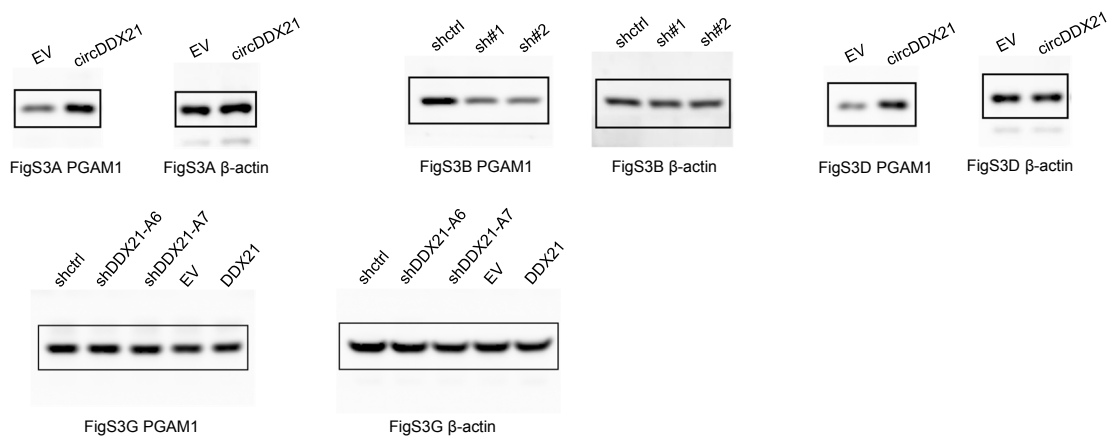

Fig. S4

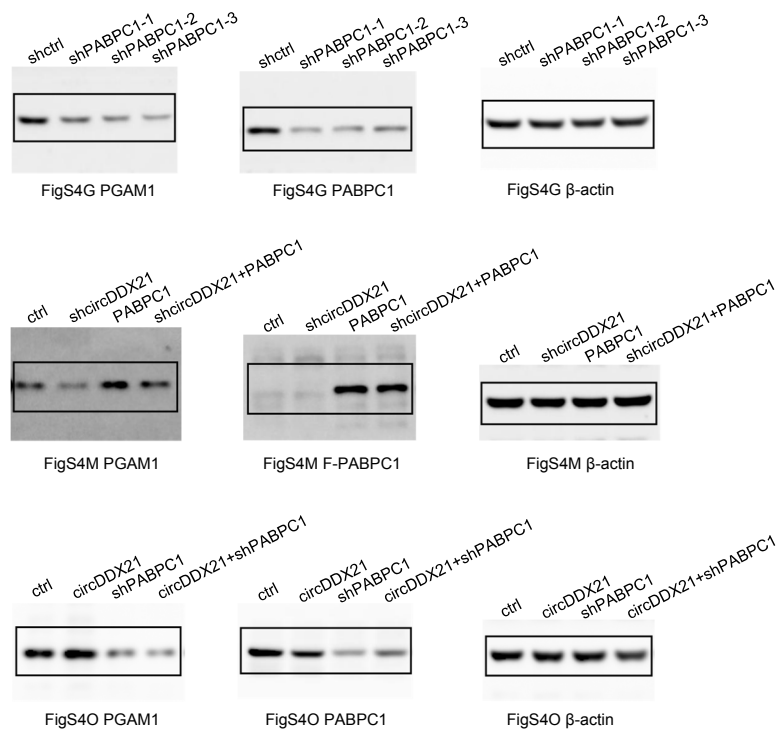

Fig. S5

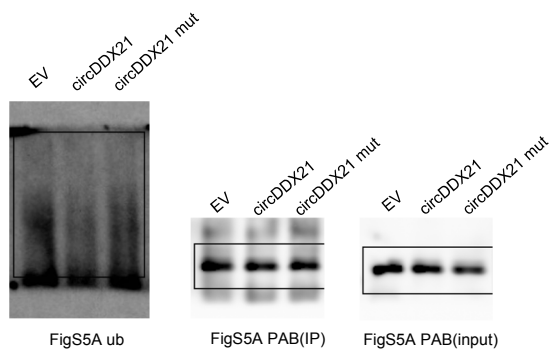

Fig. S6

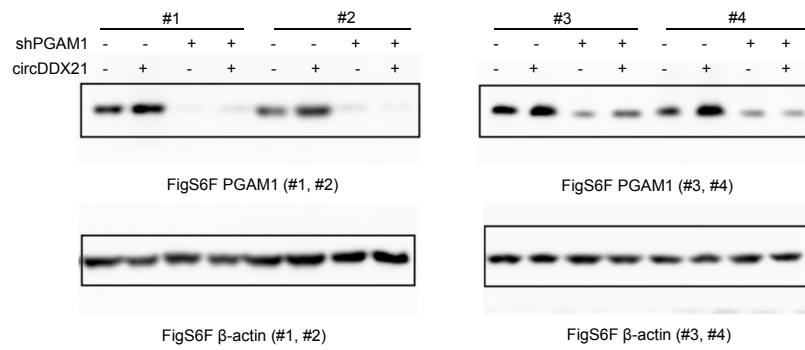

Supplement: Supplementary file 2 — Uncropped original western blots [file 41419_2024_6743_MOESM2_ESM.pdf]
